# Isolation of Three New Diketopiperazine Alkaloids from *Penicillium* sp. SCH3-Sd2

**DOI:** 10.3390/molecules31010149

**Published:** 2026-01-01

**Authors:** Eun-Young Lee, Qian Gao, Hui Tan, Prima F. Hillman, Dawoon Chung, Grace Choi, Hiyoung Kim, Kyung-Min Lim, Sang-Jip Nam

**Affiliations:** 1 College of Pharmacy, Ewha Womans University, Seoul 03760, Republic of Korea; younglee0124@naver.com; 2 Department of Chemistry and Nanoscience, Ewha Womans University, Seoul 03760, Republic of Korea; iris-gao@naver.com (Q.G.); 247878kobe@naver.com (H.T.); 3 Department of Chemistry, Faculty of Mathematics and Natural Sciences, Universitas Andalas, Kampus Limau Manis, Padang 25163, Indonesia; prima.fitria@sci.unand.ac.id; 4 Department of Biological Application & Technology, National Marine Biodiversity Institute of Korea (MABIK), Seocheon 33662, Republic of Korea; dwchung@mabik.re.kr; 5 Department of Marine Ecological Biotechnology, Kunsan National University, Gunsan 54150, Republic of Korea; gchoi@kunsan.ac.kr; 6 Department of Biomedical Science and Engineering, Konkuk University, Seoul 05029, Republic of Korea; reihyoung@konkuk.ac.kr; 7 Graduate Program in Innovative Biomaterials Convergence, Ewha Womans University, Seoul 03760, Republic of Korea

**Keywords:** *Penicillium* sp., spirocyclic diketopiperazine, Marfey’s method

## Abstract

Intensive analysis of the culture broth of the marine-derived *Penicillium* sp. strain SCH3-Sd2 led to the isolation of one spirocyclic diketopiperazine alkaloid, spirotryprostatin H (**1**), as well as two indolyl diketopiperazines, fumitremorgins O and P (**2** and **3**). The planar structures of **1**–**3** were elucidated by comprehensive spectroscopic analyses, while their absolute configurations were determined by combined NOESY and ECD analyses, and the configuration of the proline moiety was further confirmed using the advanced Marfey’s method. The evaluation of the antibacterial activity of compounds **1**–**3** against six bacterial strains revealed weak and selective activity only against *Kocuria rhizophila* KCTC 1915, with minimum inhibitory concentration (MIC) values of 64, 128, and 64 μg/mL, respectively.

## 1. Introduction

Fungi exhibit remarkable adaptability, which enables them to colonize diverse habitats, including marine, freshwater, and terrestrial environments [1,2]. Their ability to withstand extreme environmental conditions has driven the evolution of unique metabolic pathways in marine fungal species [3]. Secondary metabolites produced through these specialized metabolic pathways show promising applications in drug discovery [4]. Notably, fungi belonging to the genus *Penicillium* are commonly found in marine environments, and secondary metabolites produced by these strains have been shown to possess a broad array of biological activities [5]. In particular, indole diketopiperazine alkaloids from *Penicillium*, recognized as privileged structures, show significant biological activities, including antimicrobial, antiviral, anticancer, immunomodulatory, antioxidant, and insecticidal effects [6,7,8].

In typical laboratory conditions, the inefficient production of bioactive compounds by marine fungi is attributed to the quiescence of numerous biosynthetic gene clusters [2]. Recent developments in cultivation-based strategies aim to activate these silent gene clusters [9]. Variations in cultivation parameters, including in nutrient composition, temperature, salinity, aeration, and even the geometry of culture flask, have been employed to induce the production and facilitate the discovery of novel natural products [10]. This approach, known as the one strain−many compounds (OSMAC) strategy, facilitates the exploration of the diverse variety of complex chemical structures hidden within the specific strains [11].

Upon incorporating the OSMAC strategy into our ongoing bioprospecting effects for novel bioactive natural products from marine fungi, the cultivation of *Penicillium* sp. SCH3-Sd2 in potato dextrose broth (PDB) under static culture conditions yielded a markedly different metabolite profile compared to those of strain grown under shaking conditions. This finding prompted an in-depth investigation into the chemical constituents using high-performance liquid chromatography (HPLC) chemical profiling and nuclear magnetic resonance (NMR) analyses. Subsequent studies resulted in the isolation of spirotryprostatin H (**1**) from a 7-day shake culture, while fumitremorgin O and P (**2** and **3**) were obtained from a 21-day static culture. Herein, we report on the isolation, structures, and bioactivities of these compounds.

**Figure 1:**
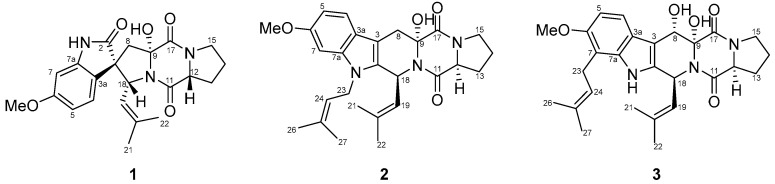
Structures of compounds **1**–**3**.

## 2. Results and Discussion

### 2.1. Structure Elucidation

Spirotryprostatin H (**1**) was isolated as a pale-yellow powder, and its molecular formula was determined as C_22_H_25_N_3_O_5_ on the basis of a protonated adduct observed at *m/z* 410.1726 [M−H]^−^ (calcd for C_22_H_24_N_3_O_5_, 410.1721) in high-resolution electrospray ionization mass spectroscopy (HR-ESI-MS). The ^1^H NMR spectroscopic data displayed three aromatic protons H-4 (*δ*_H_ 6.99, d, *J* = 8.3 Hz), H-5 (*δ*_H_ 6.50, dd, *J* = 8.3, 2.4 Hz), H-7 (*δ*_H_ 6.40, d, *J* = 2.4 Hz), one methoxy group 6-OMe (*δ*_H_ 3.72, s), and two methyl groups H-21 (*δ*_H_ 1.16, s) and H-22 (*δ*_H_ 1.49, s). ^13^C and HSQC NMR spectra revealed the presence of three aromatic methine carbons C-4 (*δ*_C_ 126.8), C-5 (*δ*_C_ 106.7), C-7 (*δ*_C_ 96.5); methoxy carbon 6-OMe (*δ*_C_ 55.2), and two methyl carbons C-21 (*δ*_C_ 17.7) and C-22 (*δ*_C_ 25.1). Further analysis of the ^13^C and HSQC NMR data enabled the assignment of four methylene groups C-8 (*δ*_C_ 41.2)/H-8 (*δ*_H_ 2.35, d, *J* = 15.0 Hz and 2.89, d, *J* = 15.0 Hz), C-13 (*δ*_C_ 27.8)/H-13 (*δ*_H_ 1.89, m and 2.22, m), C-14 (*δ*_C_ 22.7)/H-14 (*δ*_H_ 1.90, m), and C-15 (*δ*_C_ 44.8)/H-15 (*δ*_H_ 3.43, m), methine groups C-12 (*δ*_C_ 60.1)/H-12 (*δ*_H_ 4.44, m), C-18 (*δ*_C_ 60.9)/H-18 (*δ*_H_ 4.79, d, *J =* 9.0 Hz), and C-19 (*δ*_C_ 121.8)/H-19 (*δ*_H_ 4.92, d, *J* = 9.0 Hz), as well as three carbonyl groups C-2 (*δ*_C_ 182.3), C-11 (*δ*_C_ 168.2), and C-17(*δ*_C_ 164.3). These NMR data indicated that **1** contains a skeleton similar to the oxindole diketopiperazine structure observed in spirotryprostatin A [12], and asperdiketopoid D [13], except for the presence of a hydroxyl group at 9-OH (*δ*_H_ 7.42, s)/C-9 (*δ*_C_ 89.4) (Table 1, Figure 1). Compound **1** shares the same indole diketopiperazine core as spiro spirotryprostatin A and asperdiketopoid D; however, a clear structural difference is observed at the C-9 position. In spirotryprostatin A and asperdiketopoid D, C-9 is a methine carbon, whereas in compound **1**, C-9 is an oxygenated quaternary carbon (*δ*_C_ 89.4) bearing a hydroxy group. This difference is supported by the absence of C-9 proton signal in ^1^H NMR spectrum of **1** and the corresponding downfield shift in the ^13^C NMR data (Table 1).

**Figure 2:**
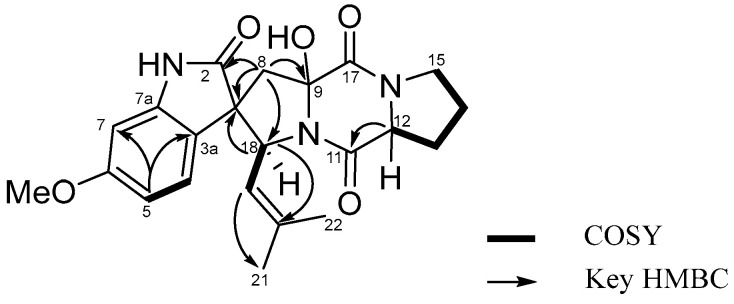
COSY and key HMBC correlations of **1**.

The analysis of COSY, HSQC and HMBC NMR spectroscopic data enabled the construction of distinct fragments with a unique structural skeleton, characterized by a spiro ring system. This system consists of a γ-lactam fused to a benzene ring and a pentacyclic enamine linked to a diketopiperazine moiety. The diketopiperazine moiety, linked to the pentacyclic enamine derived from a proline residue and to an isoprenyl group, was assigned based on the long-range HMBC correlations from 9-OH (*δ*_H_ 7.42, s) to C-9 (*δ*_C_ 89.4) and C-17 (*δ*_C_ 164.3), from H-8 (*δ*_H_ 2.35, d, *J* = 15.0 Hz and 2.89, d, *J* = 15.0 Hz) to C-2 (*δ*_C_ 182.3), C-3 (*δ*_C_ 55.9), C-3a (*δ*_C_ 119.8), C-9 (*δ*_C_ 89.4), and C-18 (*δ*_C_ 60.9), and from H-12 (*δ*_H_ 4.44, m) to C-11 (*δ*_C_ 168.2), and C-13 (*δ*_C_ 27.8). The isoprenyl group was characterized by the COSY correlations H-18 (*δ*_H_ 4.79, d, *J =* 9.0 Hz)/H-19 (*δ*_H_ 4.92, d, *J =* 9.0 Hz), combined with the HMBC correlations from H-18 (*δ*_H_ 4.79, d, *J =* 9.0 Hz) to C-2 (*δ*_C_ 182.3), C-3 (*δ*_C_ 55.9), C-8 (*δ*_C_ 41.2), C-19 (*δ*_C_ 121.8), C-20 (*δ*_C_ 135.2), from H-19 (*δ*_H_ 4.92, d, *J =* 9.0 Hz) to C-21 (*δ*_C_ 17.7) and C-22 (*δ*_C_ 25.1), from H-21 (*δ*_H_ 1.16, s) to C-19 (*δ*_C_ 121.8), C-20 (*δ*_C_ 135.2), C-22 (*δ*_C_ 25.1), and from H-22 (*δ*_H_ 1.49, s) to C-19 (*δ*_C_ 121.8), C-20 (*δ*_C_ 135.2), and C-21 (*δ*_C_ 17.7) (Figures S1–S7). These results illustrate the connectivity of the spiro ring system, which comprised a γ-lactam fused to a benzene ring and a pentacyclic enamine associated with the diketopiperazine moiety. Thus, the planar structure of **1** was assigned as shown in Figure 2.

**Figure 3:**
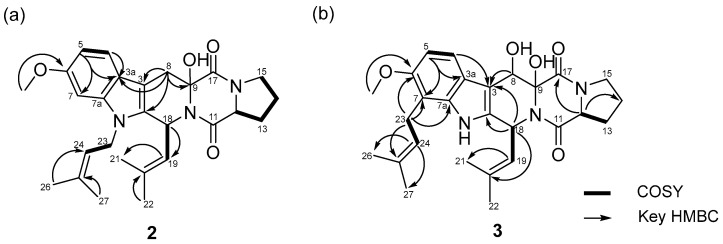
COSY and key HMBC correlations of **2** (**a**) and **3** (**b**).

Fumitremorgin O (**2**), obtained as a pale-yellow amorphous powder, possessed a molecular formula of C_27_H_33_N_3_O_4_, as determined from its HRESIMS ion peaks at *m*/*z* 464.2542 [M+H]^+^ (calcd for C_27_H_34_N_3_O_4_, 464.2544). Both the UV and NMR spectra consistently confirmed the presence of an indole diketopiperazine system within compound **2** [14]. The ^1^H NMR spectrum of **2** indicated the presence of one methoxyl proton 6-OMe (*δ*_H_ 3.76, s), four methyl protons H-21 (*δ*_H_ 1.91, s), H-22 (*δ*_H_ 1.59, s), H-26 (*δ*_H_ 1.85, s),and H-27 (*δ*_H_ 1.66, s), five sets of methylene protons H-8 (*δ*_H_ 3.08, d, *J* = 16.0 Hz and 3.40 d, *J* = 16.0 Hz), H-13 (*δ*_H_ 1.85, m and 2.27, m), H-14 (*δ*_H_ 1.86, m), H-15 (*δ*_H_ 3.45, m), and H-23 (*δ*_H_ 4.54, m and 4.64, m), and four methine protons H-12 (*δ*_H_ 4.37, m), H-18 (*δ*_H_ 5.93 d, *J* = 9.8 Hz), H-19 (*δ*_H_ 4.82, d, *J* = 9.8 Hz), H-24 (*δ*_H_ 4.98, m), and three aromatic protons H-4 (*δ*_H_ 7.42, d, *J* = 8.2 Hz), H-5 (*δ*_H_ 6.69, dd, *J* = 8.3 and 2.2 Hz), and H-7 (*δ*_H_ 6.80, d, *J* = 2.2 Hz). The ^13^C NMR and HSQC spectra displayed 27 carbon signals, including eight indole carbons C-2 (*δ*_C_ 132.3), C-3 (*δ*_C_ 103.3), C-3a (*δ*_C_ 121.1), C-4 (*δ*_C_ 118.4), C-5 (*δ*_C_ 108.5), C-6 (*δ*_C_ 155.3), C-7 (*δ*_C_ 93.5), and C-7a (*δ*_C_ 137.0), one methoxy carbon 6-OMe (*δ*_C_ 55.0), four methyl carbons C-21 (*δ*_C_ 17.7), C-22 (*δ*_C_ 25.1), C-26 (*δ*_C_ 18.0), and C-27 (*δ*_C_ 25.2), five methylene carbons C-8 (*δ*_C_ 29.4), C-13 (*δ*_C_ 28.5), C-14 (*δ*_C_ 22.1), C-15 (*δ*_C_ 44.9), and C-23 (*δ*_C_ 41.0), four methine carbons C-12 (*δ*_C_ 57.9), C-18 (*δ*_C_ 40.7), C-19 (*δ*_C_ 123.1), and C-24 (*δ*_C_ 120.4), two carbonyl carbons C-11 (*δ*_C_ 164.9) and C-17 (*δ*_C_ 169.9), and four quaternary carbons C-9 (*δ*_C_ 82.9), C-11 (*δ*_C_ 164.9), C-20 (*δ*_C_ 132.9), and C-25 (*δ*_C_ 133.7). Thorough analysis of the ^1^H and ^13^C NMR data (Table 1 and Table 2), combined with the COSY and HMBC correlations (Figure 3), revealed that **2** shared the same planar structure as the prenylated indole diketopiperazine alkaloid fumitremorgin B [15], except for the presence of a methylene group at H-8 (*δ*_H_ 3.08, d, *J* = 16.0 Hz and 3.40, d, *J* = 16.0 Hz)/C-8 (*δ*_C_ 29.4). This substitution was confirmed by the HMBC correlations from H-8 (*δ*_H_ 3.08, d, *J* = 16.0 Hz and 3.40, d, *J* = 16.0 Hz) to C-2 (*δ*_C_ 132.3), C-3 (*δ*_C_ 103.3), C-3a (*δ*_C_ 121.1), and C-9 (*δ*_C_ 82.9) (Figures S9–S15). In addition, the methylene carbon C-23 exhibited a characteristic downfield chemical shift at *δ*_C_ 41.0, which is consistent with a nitrogen-adjacent methylene (N–CH_2_–) environment. This observation strongly supports the assignment of the isoprenyl unit being directly attached to the indole nitrogen (N-1), despite the weak or ambiguous long-range HMBC correlations involving H_2_-23. Taken together, the spectroscopic data led to the definitive planar structural assignment of **2** (Figure 3a).

Fumitremorgin P (**3**) was isolated as a pale-yellow amorphous powder. Its molecular formula was determined to be C_27_H_33_N_3_O_5_ based on the HRESIMS ion peaks at *m*/*z* 480.2495 [M+H]^+^ (calcd for C_27_H_34_N_3_O_5_, 480.2493). Detailed analysis of the NMR data (Figures S17–S23 and Table 1) indicated that compound **3** contained an indole diketopiperazine system similar to fumitremorgin B (**7**) except for the presence of an isoprenyl unit [C-23 (*δ*_C_ 23.4) to C-27 (*δ*_C_ 25.2)] attached to the indole C-7 (*δ*_C_ 111.9). Additionally, the ^1^H NMR spectrum displayed an additional hydroxyl group C-8 (*δ*_C_ 67.8). The key HMBC correlations from H-23 (*δ*_H_ 3.41, m and 3.54, m) to C-6 (*δ*_C_ 152.2), C-7 (*δ*_C_ 111.9), C-7a (*δ*_C_ 136.5), and C-24 (*δ*_C_ 123.1) confirmed the location of the isoprenyl unit [C-23 (*δ*_C_ 23.4) to C-27 (*δ*_C_ 25.2)] and allowed to assign the complete planar structure of **3** (Figure 3b). Comparison of compounds **2** and **3** revealed that, while both share a fumitremorgin-type indole diketopiperazine framework, they differ in their prenylation and hydroxylation patterns. Compound **2** possesses a methylene group at C-8 and an N-1-linked isoprenyl unit, whereas compound **3** features an additional hydroxy group at C-8 and a differently positioned isoprenyl unit attached to the indole C-7, as supported by key HMBC correlations. These structural features clearly distinguish compounds **2** and **3** from each other and from previously reported fumitremorgin analogues.

Spirotryprostatin H (**1**) and fumitremorgins O (**2**) and P (**3**) expand the chemical space of prenylated indole diketopiperazines, a well-recognized class of secondary metabolite produced by *Aspergillaceae* [16]. Compared to previously reported congeners, the presence of the 9-hydroxy-substituted oxindole system in **1** is uncommon within this scaffold, suggesting that late-stage, enzyme-mediated oxidation reactions substantially contribute to structural diversification in this biosynthetic pathway [17,18]. Similarly, the distinct prenylation patterns observed for **2** and **3** further highlight the flexibility of DKP cyclization and subsequent post-NRPS modifications.

Taken together, these findings show that marine-derived fungal strains continue to generate unique structural variations within the spirotryprostatin and fumitremorgin lineages. Such structural diversity, arising from oxidative and prenylation-driven modifications, provides a broader platform for the exploration of structure–activity relationship in prenylated indole DKPs.

### 2.2. Absolute Configurations of Compounds **1**–**3**

**Figure 4:**
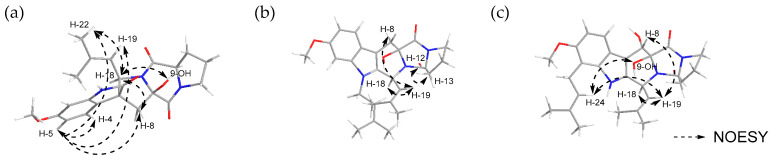
NOESY correlation of (**a**) spirotryprostatin H (**1**), (**b**) fumitremorgin O (**2**), and (**c**) fumitremorgin P (**3**).

The relative stereochemistry of compounds **1**–**3** was determined through NOESY experiments. For compound **1**, NOESY correlations were observed between H-4 (*δ*_H_ 6.99) and H-5 *(δ*_H_ 6.50), H-8 (*δ*_H_ 2.35 and 2.89), H-19 (*δ*_H_ 4.92), and H-22 (*δ*_H_ 1.49), between H-18 (*δ*_H_ 4.79) and H-8 (*δ*_H_ 2.35 and 2.89), H-21 (*δ*_H_ 1.16) and H-22 (*δ*_H_ 1.49), between H-19 (*δ*_H_ 4.92) and H-8 (*δ*_H_ 2.89), H-21 (*δ*_H_ 1.16), and H-22 (*δ*_H_ 1.49), as well as between 9-OH (*δ*_H_ 7.42) and H-12 (*δ*_H_ 4.44)/H-18 (*δ*_H_ 4.79). This indicated that these protons resided on the same face, which allowed the relative configuration as 9R*, 18R* (Figure 4a). For compound **2**, the NOESY correlations observed between H-12 (*δ*_H_ 4.37) and H-13 (*δ*_H_ 2.27), between H-19 (*δ*_H_ 4.82) and H-8 (*δ*_H_ 3.08), as well as between H-18 (*δ*_H_ 5.93) and H-19 (*δ*_H_ 4.82) indicated that these protons were located on the same face, enabling the relative configuration to be assigned as 9R*, 18S* (Figure 4b). In the case of compound **3**, NOESY correlations were established between H-18 (*δ*_H_ 5.83) and H-19 (*δ*_H_ 4.78), between H-8 (*δ*_H_ 5.51) and H-19 (*δ*_H_ 4.78), and H-24 (*δ*_H_ 5.20), between 9-OH (*δ*_H_ 4.09) and H-24 (*δ*_H_ 5.18), as well as between 8-OH (*δ*_H_ 4.76) and H-18 (*δ*_H_ 5.83), supporting the assignment of 8R*, 9S*, 18R* as the relative configuration of **3** (Figure 4c).

The absolute configurations of proline (Pro) moiety in compounds **1**–**3** were determined through acid hydrolysis, followed by the advanced Marfey’s method [19]. The retention times of the 1-fluoro-2,4-dinitrophenyl-5-_L_-leucineamide (_L_-FDLA) derivatives of the constituent proline residues in **1**–**3** were evaluated using LC-ESI-MS. The Pro amino acid in compound **1** was identified as possessing a _D_-configuration, meaning the *R* configuration at C-12 (Figure S26), whereas those in compounds **2** and **3** were determined to a possess an _L_-configuration, meaning the *S* configuration at C-12 (Figure S27).

**Figure 5:**
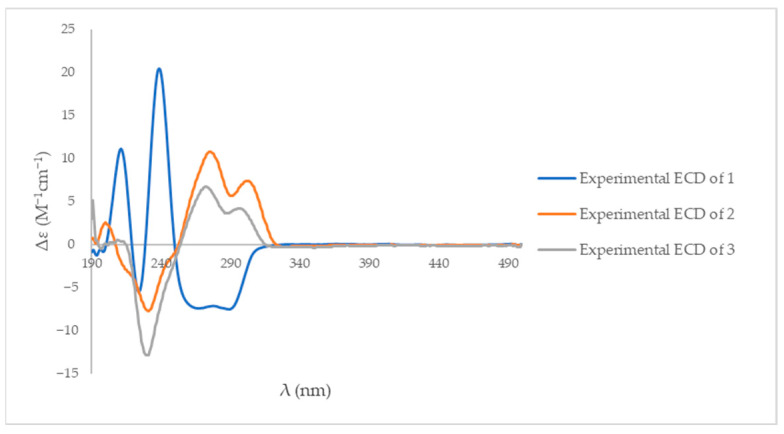
The experimental ECD spectra of compounds **1**–**3** in methanol.

The ECD spectrum of compound **1** displayed a negative Cotton effect with a minimum at 282 nm and a positive Cotton effect with a maximum at 242 nm (Table S1). Compounds **2** and **3** exhibited negative Cotton effects with minima at 229 nm, while positive Cotton effects were observed with maxima at 270 nm for compound **2** and 292 nm for compound **3** (Table S1). Compared with previously reported ECD data for structurally related spirotryprostatin [20,21,22] and fumitremorgin [8,20] analogues, these Cotton effect patterns were consistent with a 3*R* configuration for compound **1** and supported the stereochemical assignments for compounds **2** and **3** (Figure 5).

Based on the NOESY spectra, the comparison with the ECD data of reported analogues, and the advanced Marfey’s method, the absolute configuration of spirotryprostatin H (**1**) was assigned as 3*R,* 9*R*, 18*R*, 12*R*, whereas that of fumitremorgins O (**2**) and P (**3**) was assigned as 9*R*, 18*S*, 12*S* and 8*S*, 9*R*, 18*S*, 12*S*, respectively.

### 2.3. Antibacterial Activity

The antibacterial activities of compounds **1**–**3** were evaluated against three Gram-positive bacteria (*Bacillus subtilis* KCTC 1021, *Kocuria rhizophila* KCTC 1915, and *Staphylococcus aureus* KCTC 1621) and three Gram-negative bacteria (*Escherichia coli* KCTC 2441, *Salmonella typhimurium* KCTC 2515, and *Klebsiella pneumoniae* KCTC 2690). Among the tested strains, compounds **1**–**3** displayed weak antibacterial effects only against *Kocuria rhizophila* KCTC 1915, with a minimum inhibitory concentration (MIC) of 64, 128, 64 μg/mL, respectively (Table 2). In contrast, no significant inhibitory effects were observed against the other bacterial strains, indicating that the present compounds possessed selective and weak antibacterial activity

The weak antibacterial activity of compounds **1**–**3**, which was only observed against the Gram-positive strain *K. rhizophila*, may be attributed to structural factors such as low membrane permeability or limited interaction with bacterial targets. This lack of efficacy against Gram-negative bacteria is consistent with previous findings that the outer membrane of Gram-negative species serves as a permeability barrier, limiting the entry of indole-based metabolites and related compounds [23,24,25]. This selectivity suggests that the present compounds are not broad-spectrum antibiotics, but could serve as scaffolds for structural modifications to enhance the antibacterial of a drug.

**Table 2:** Antibacterial activity (MIC, μg/mL) of compounds **1**–**3** ^a^.

Minimal Inhibitory Concentration (μg/mL)						
Compounds	Gram (+) Bacteria			Gram (−) Bacteria		
	*B. subtillis* KCTC 1021	*K. rhizophila* KCTC 1915	*S. aureus* KCTC 1621	*E. coli* KCTC 2441	*S. tyhimurium* KCTC 2515	*K. pneumonia* KCTC 2690
**1**	>128	64	>128	>128	>128	>128
**2**	>128	128	>128	>128	>128	>128
**3**	>128	64	>128	>128	>128	>128
Ampicillin	0.5	0.25	2	64	16	>128
Vancomycin	0.25	0.25	0.25	>128	>128	>128

^a^ Each sample was tested in triplicate.

## 3. Materials and Methods

### 3.1. General Experimental Procedures

Optical rotations were determined using a Krüss Optronic P-8000 polarimeter with a 5-cm cell (Krüss, Hambrug, Germany) in methanol (MeOH). UV spectra were acquired with a Varian Cary 50 UV–vis spectrophotometer (Varian Inc., Mulgrave, Australia), whereas ECD spectra were obtained using a JASCO J-810 spectrometer (JASCO Inc., Tokyo, Japan). IR spectra were recorded on a Thermo Fisher Scientific Nicolet iS 10 FT-IR spectrometer (Waltham, MA, USA). The weak absorption observed around 2200–2400 cm^−1^ was also present in the CHCl_3_ blank spectrum and was therefore attributed to background signals rather than the sample (Figure S25). NMR spectra were obtained utilizing a Varian Inova spectrometer (Varian Medical Systems, Inc., Charlottesville, VA, USA) operating at 500 MHz for ^1^H and 125 MHz for ^13^C experiments, and a Bruker NMR spectrometer (Bruker, Middlesex, MA, USA) working at 300 MHz for ^1^H measurements. The residual solvent signals served as internal references, with *δ*_H_ 3.31 ppm and *δ*_C_ 49.0 ppm for deuterated methanol (CD_3_OD), and *δ*_H_ 7.26 and 1.56 ppm for chloroform-d (CDCl_3_). Low-resolution LC-MS data were measured using the Agilent Technologies 1260 quadrupole (Agilent Technologies, Santa Clara, CA, USA) and Waters Micromass ZQ LC/MS system with a reversed-phase column (Phenomenex Luna C18 (2), 100 Å, 100 mm × 4.6 mm, 5 µm), operating at a flow rate of 1.0 mL/min, at the National Research Facilities and Equipment Center (NanoBioEnergy Materials Center) at Ewha Womans University. Column chromatography (CC) experiment was performed using a reversed-phase C18 gel (70-230 mesh, Merck, Darmstadt, Germany) with a step-gradient solvent of water (H_2_O) and methanol (MeOH). Subsequently, the fractions were purified using a reversed-phase HPLC (Phenomenex Luna C-18 (2), 100 Å, 250 × 10 mm, 2.0 mL/min, 5 μm) (Phenomenex, Torrance, CA, USA). The minimum inhibitory concentration (MIC) of the antibacterial test was determined by measuring the optical density at 600 nm (OD_600_) for various sample dilutions using a SpectraMax^®^ ABS Plus microplate reader (Molecular Devices, San Jose, CA, USA).

### 3.2. Collection and Identification of the Strain SCH3-Sd2

Marine sediment was collected on June 15, 2020, from Suncheon, Jeollanam-do, Republic of Korea (34°50′47.9″ N; 127°31′28.3″ E). A 100 μL aliquot of the sediment suspension, prepared by suspending the sample in 1 × PBS solution, was spread onto potato dextrose agar (PDA, BD DifcoTM, Sparks, MD, USA) plates supplemented with 3.5% (*w*/*v*) NaCl, 0.01% (*w*/*v*) ampicillin, and 0.01% (*w*/*v*) streptomycin. The plates were incubated at 20 °C for 7 days. After incubation, the developed colonies were repeatedly transferred onto fresh media to obtain pure isolates. The isolates were preserved in 20% glycerol solution at −80 °C.

One of the isolates, designated SCH3-Sd2, was identified based on the nucleotide sequence of the β-tubulin gene marker (Figure S28). The strain was first inoculated into potato dextrose broth (PDB; BD, USA) and incubated at 25 °C for 3 days to obtain mycelia. Genomic DNA was extracted from the harvested mycelia using the phenol-chloroform method [26]. The β-tubulin fragment was amplified by polymerase chain reaction (PCR) using the primers Bt2a and Bt2b [27]. The resulting 415 bp PCR product was purified using a PCR purification kit (Qiagen, Hilden, Germany) and sequenced by Macrogen Inc. (Seoul, Republic of Korea).

The β-tubulin sequence of SCH3-Sd2 was used as a query for a BLAST search (https://blast.ncbi.nlm.nih.gov/Blast.cgi) (accessed on 10 October 2025) in the NCBI database to assess its homology with closely related fungal species. Based on the homology analysis, the β-tubulin sequence of SCH3-Sd2 showed 99.76% identity with that of the *Penicillium caprifimosum* strain MGS 2017. As a result, SCH3-Sd2 was identified as *P. caprifimosum*. This strain has been deposited in the Marine BioBank database of the National Marine Biodiversity Institute of Korea (MABIK), under accession number MABIK FU00001487.

### 3.3. Fermentation, Extraction, and Isolation

The strain SCH3-Sd2 underwent two distinct culturing procedures. First, it was cultivated under shaking conditions in 10 L of 2.5 L Ultra Yield^®^ flasks (Thomson Instrument Company, Oceanside, CA, USA) with 1 L of PDB SW medium (24 g/L of PDB and 37.8 g/L of sea salt in 1 L of distilled water), followed by shaking at 120 rpm and 27 °C. After 7 days of cultivation, the broth was extracted using ethyl acetate (EtOAc) to produce 10 L of extract, and the soluble fraction was further concentrated *in vacuo* to obtain 2.5 g of crude extract. The crude extract was subjected to flash column chromatography on a C18 resin eluted with H_2_O/CH_3_OH (80/20, 60/40, 50/50, 40/60, 30/70, 20/80, 0/100, 200 mL each) to obtain seven fractions (F1–F7). Then, fraction 4 (166.5 mg) underwent purification via reversed-phase HPLC (Phenomenex Luna C-18 (2), 100 Å, 250 × 10 mm, 2.0 mL/min, 5 μm, UV = 254 nm) using 30% aqueous CH_3_CN containing 0.1% trifluoroacetic acid (TFA), yielding spirotryprostatin H (**1**, 2.9 mg, *t*_R_ = 41.0 min).

In the subsequent culture, SCH3-Sd2 was statically cultured in 18 L of 2.5 L Ultra Yield^®^ flasks containing 1 L of PDB SW medium for 21 days at 27 °C. After cultivation and extraction using EtOAc (18 L extract overall), the soluble fraction was subsequently concentrated to yield 7.0 g of crude extract. Moreover, fraction 6 (214.5 mg) underwent further purification by reversed-phase HPLC (Phenomenex Luna C-18 (2), 100 Å, 250 × 10 mm, 2.0 mL/min, 5 μm, UV = 254 nm) under isocratic conditions using a 50% aqueous CH_3_CN containing 0.1% TFA as the mobile phase. The procedure performed under either low-light or dark conditions led to the isolation of fumitremorgin O (**2**, 4.7 mg, *t*_R_ = 60.5 min) and fumitremorgin P (**3**, 11.2 mg, *t*_R_ = 52.5 min).

*Spirotryprostatin H (***1***)*: pale-yellow powder; ${\left[\alpha \right]}_{D}^{21}$ = −97 (c 0.05, chloroform); UV (MeOH) max (log ε) 201 (3.17), 219 (3.16), and 298 (2.10) nm; IR (KBr) ν_max_ 3595, 2955, 2939, 1717, 1684, 1653, 1540, 1376 and 1061 cm^−1^; ^1^H and ^13^C NMR data (400 and 100 MHz, DMSO-*d*_6_), Table 1; HRESIMS *m*/*z* 410.1726 [M−H]^−^ (calcd for C_22_H_24_N_3_O_5_, 411.1721).

*Fumitremorgin O (***2***)*: pale-yellow amorphous powder; ${\left[\alpha \right]}_{D}^{21}$ = +128 (c 0.05, chloroform); UV (MeOH) max (log ε) 217 (3.26), 276 (1.78), and 295 (1.71) nm; IR (KBr) ν_max_ 2955, 2924, 1653, 1458, 1379, 1225, 1172, and 1083 cm^−1^; ^1^H and ^13^C NMR data (400 and 100 MHz, DMSO-*d*_6_), Table 1; HRESIMS *m*/*z* 464.2542 [M+H]^+^ (calcd for C_27_H_34_N_3_O_5_, 464.2544).

*Fumitremorgin P (***3***)*: pale-yellow amorphous powder; ${\left[\alpha \right]}_{D}^{21}$ = +128 (c 0.05, chloroform); UV (MeOH) max (log ε) 202 (3.20), 220 (3.17), and 298 (2.66) nm; IR (KBr) ν_max_ 3597, 2954, 1718, 1686, 1653, 1506, 1457, 1209 and 1079 cm^−1^; ^1^H and ^13^C NMR data (400 and 100 MHz, DMSO-*d*_6_), Table 1; HRESIMS *m*/*z* 480.2495 [M+H]^+^ (calcd for C_27_H_34_N_3_O_4_, 480.2493).

### 3.4. Acid Hydrolysis and Advanced Marfey’s Analysis of Spirotryprostatin H

The absolute configurations of spirotryprostatin H (**1**), fumitremorgin O (**2**), and fumitremorgin P (**3**) were determined using the advanced Marfey’s method. Samples of compounds **1**–**3** (1 mg each) were dissolved in 100 μL of 2 *N* HCl and heated at 100 °C for 12 h. The resulting hydrolysate was evaporated to dryness under a stream of N_2_ gas; the residue was subsequently redissolved in distilled H_2_O and divided into two aliquots. After removing water under a continuous N_2_ stream, each hydrolysate was dissolved in 100 μL of 1 *N* NaHCO_3_ and derivatized with 100 μL of 1% _L_-FDLA (1-fluoro-2,4-dinitrophenyl-5-_L_-leucine amide) in acetone. The reaction mixtures were incubated at 65 °C for 1 h and subsequently quenched with 2 *N* HCl. A small volume of CH_3_CN (100 μL) was added to the reaction products and injected into the LC-ESI-MS system with following chromatographic conditions: Phenomenex Luna C18 (2) 100 Å, 100 mm × 4.6 mm, 5 μm, 1 mL/min flow, solvent A (0.01% formic acid in H_2_O), solvent B (0.01% formic acid in CH_3_CN), A/B = 95:5 → 50:50 (50 min) → 100:0 (70 min) → 0:100 (85 min) → 95:5 (90 min). The standard amino acids were prepared according to an established protocol and subsequently analyzed. The reaction products were analyzed using UV spectroscopy at 340 nm in positive ESIMS mode [28].

### 3.5. Antibacterial Activity Assay

Antibacterial susceptibility assays were conducted against six pathogenic bacterial strains: *Escherichia coli* (KCTC 2441), *Salmonella typhimurium* (KCTC 2515), *Klebsiella pneumoniae* (KCTC 2690), *Bacillus subtilis* (KCTC 1021), *Kocuria rhizophila* (KCTC 1915), and *Staphylococcus aureus* (KCTC 1621). Bacterial cultures were grown overnight in Mueller-Hinton broth (MHB) at 37 °C. The bacterial suspensions were subsequently adjusted to match the 0.5 McFarland standard (approximately 1.0 × 10^8^ CFU/mL) and further diluted to achieve a final inoculum concentration of 5.0 × 10^5^ CFU/mL.

The test compounds and positive controls (ampicillin and vancomycin) were initially dissolved in dimethyl sulfoxide (DMSO) to prepare stock solutions with a concentration of 10 mg/mL. These stock solutions were further diluted in MHB to achieve a working concentration of 256 μg/mL. A 100 μL aliquot of each prepared sample was added to the first column of a sterile 96-well microtiter plate, followed by twofold serial dilutions across the plate to achieve a concentration range of 128–0.25 μg/mL. Then, 50 μL of the standardized bacterial suspension was added to each well, resulting in final test concentrations of 128, 64, 32, 16, 8, 4, 2, 1, 0.5, and 0.25 μg/mL.

The plates were incubated at 37 °C for 18–24 h under static conditions. The MIC was defined as the lowest concentration of the compound that completely inhibited visible bacterial growth. Growth inhibition was confirmed by measuring the absorbance at 600 nm using a SpectraMax^®^ ABS Plus microplate reader (Molecular Devices, San Jose, CA, USA). All experiments were performed in triplicate to ensure reproducibility.

## 4. Conclusions

In conclusion, three prenylated indole diketopiperazines, spirotryprostatin H (**1**), fumitremorgin O (**2**) and fumitremorgin P (**3**), were isolated from marine-derived *Penicillium* sp. SCH3-Sd2. Their planar structures and absolute configurations were established via a comprehensive spectroscopic analysis, including NOESY, ECD, and the advanced Marfey’s method. Although compounds **1**–**3** exhibited only weak and selective antibacterial activity, the uncommon oxidation pattern and distinct prenylation features observed in these DKPs expand the structural diversity of the spirotryprostatin or fumitremorgin family. This study highlights the potential of marine-derived fungi as a valuable source of structurally unique indole alkaloids and provides a basis for further studies of their structure–activity relationship.

## Figures and Tables

**Table 1:** NMR data for compounds **1**–**3** in DMSO-*d*_6_.

Pos.	1		2				3	
	*δ*_C_ ^a^, Mult.	*δ*_H_ ^b^ (*J* in Hz)	*δ*_C_ ^a^, Mult.	*δ*_H_ ^b^ (*J* in Hz)	COSY	HMBC	*δ*_C_ ^a^, Mult.	*δ*_H_ ^b^ (*J* in Hz)
1-NH								10.44, s
2	182.3, qC		132.3, qC				131.1, qC	
3	55.9, qC		103.3, qC				106.5, qC	
3a	119.8, qC		121.1, qC				121.5, qC	
4	126.8, CH	6.99, d (8.3)	118.4, CH	7.42, d (8.3)	5	3, 6, 7a	117.7, CH	7.56, d (8.7)
5	106.7, CH	6.50, dd (8.3, 2.4)	108.5, CH	6.69, dd (8.3, 2.2)	4, 7	4, 7	105.6, CH	6.76, d (8.7)
6	159.8, qC		155.3, qC				152.2, qC	
7	96.5, CH	6.40, d (2.4)	93.4, CH	6.80, d (2.2)		4, 5, 6	111.9, qC	
7a	142.8, qC		137.0, qC				136.5, qC	
8	41.2, CH_2_	α 2.35, d (15.0); β 2.89, d (15.0)	29.4, CH_2_	α 3.08, d (16.0); β 3.40, d (16.0)		2, 3, 3a, 9	67.8, CH	5.51, s
9	89.4, qC		82.9, qC				83.3, qC	
11	168.2, qC		164.9, qC				166.2, qC	
12	60.1, CH	4.44, m	57.9, CH	4.37, m	13	13 ^c^	58.2, CH	4.41, m
13	27.8, CH_2_	α 1.89, m; β 2.22, m	28.5, CH_2_	α 1.85, m; β 2.27, m	12, 14		28.5, CH_2_	α 1.87, m; β 2.29, m
14	22.7, CH_2_	1.90, m	22.1, CH_2_	1.86, m	13, 15		22.0, CH_2_	1.89, m
15	44.8, CH_2_	3.43, m	44.7, CH_2_	3.45, m	14		44.9, CH_2_	3.45, m
17	164.3, qC		169.9, qC				170.2, qC	
18	60.9, CH	4.79, d (9.0)	40.7, CH	5.93, d (9.8)	19	19, 20	48.7, CH	5.83, d (9.5)
19	121.8, CH	4.92, d (9.0)	123.1, CH	4.82, d (9.8)	18	21 ^c^	124.0, CH	4.78, d (9.5)
20	135.2, qC		132.9, qC				133.0, qC	
21	17.7, CH_3_	1.16, s	17.7, CH_3_	1.91, s		19, 20, 22	18.4, CH_3_	1.92, s
22	25.1, CH_3_	1.49, s	25.1, CH_3_	1.59, s		19, 20, 21	25.4, CH_3_	1.57, s
23			41.0, CH_2_	4.54, m; β 4.64, m	24	25 ^c^	23.4, CH_2_	3.54, m/3.41, m
24			120.4, CH	4.98, m	23	26 ^c^, 27 ^c^	123.1, CH	5.20, m
25			133.7, qC				130.2, qC	
26			18.0, CH_3_	1.85, s		24, 25, 27	17.9, CH_3_	1.76, s
27			25.2, CH_3_	1.66, s		24, 25, 26	25.2, CH_3_	1.62, s
6-OMe	55.2, CH_3_	3.72, s	55.0, CH_3_	3.76, s		6	56.4, CH_3_	3.76, s
8-OH								4.76, s
9-OH		7.42, s		6.53, br s				4.09, s

^a^ 400 MHz for ^1^H NMR. ^b^ 100 MHz for ^13^C NMR. ^c^ Weak HMBC signals.

## Data Availability

The data supporting the reported results are contained within the manuscript and its Supporting Information.

## Supplementary Materials

The following supporting information can be downloaded at: https://www.mdpi.com/article/10.3390/molecules31010149/s1, Figure S1: ^1^H NMR Spectrum of spirotryprostatin H (**1**) in DMSO-*d*_6_; Figure S2: ^13^C NMR Spectrum of spirotryprostatin H (**1**) in DMSO-*d*_6_; Figure S3: COSY NMR Spectrum of spirotryprostatin H (**1**) in DMSO-*d*_6_; Figure S4: HSQC NMR Spectrum of spirotryprostatin H (**1**) in DMSO-*d*_6_; Figure S5: HMBC NMR Spectrum of spirotryprostatin H (**1**) in DMSO-*d*_6_; Figure S6: NOESY NMR Spectrum of spirotryprostatin H (**1**) in DMSO-*d*_6_; Figure S7: High resolution mass data of spirotryprostatin H (**1**); Figure S8: FT-IR spectrum of spirotryprostatin H (**1**); Figure S9: ^1^H NMR Spectrum of fumitremorgin O (**2**) in DMSO-*d*_6_; Figure S10: ^13^C NMR Spectrum of fumitremorgin O (**2**) in DMSO-*d*_6_; Figure S11: COSY NMR Spectrum of fumitremorgin O (**2**) in DMSO-*d*_6_; Figure S12: HSQC NMR Spectrum of fumitremorgin O (**2**) in DMSO-*d*_6_; Figure S13: HMBC NMR Spectrum of fumitremorgin O (**2**) in DMSO-*d*_6_; Figure S14: NOESY NMR Spectrum of fumitremorgin O (**2**) in DMSO-*d*_6_; Figure S15: High resolution mass data of fumitremorgin O (**2**); Figure S16: FT-IR spectrum of fumitremorgin O (**2**); Figure S17: ^1^H NMR Spectrum of fumitremorgin P (**3**) in DMSO-*d*_6_; Figure S18: ^13^C NMR Spectrum of fumitremorgin P (**3**) in DMSO-*d*_6_; Figure S19: COSY NMR Spectrum of fumitremorgin P (**3**) in DMSO-*d*_6_; Figure S20: HSQC NMR Spectrum of fumitremorgin P (**3**) in DMSO-*d*_6_; Figure S21: HMBC NMR Spectrum of fumitremorgin P (**3**) in DMSO-*d*_6_; Figure S22: NOESY NMR Spectrum of fumitremorgin P (**3**) in DMSO-*d*_6_; Figure S23: High resolution mass data of fumitremorgin P (**3**); Figure S24: FT-IR spectrum of fumitremorgin P (**3**); Figure S25: FT-IR spectrum of CHCl_3_ background signal; Table S1: ECD data of compounds **1**–**3**; Table S2: NMR correlation data for compounds **1**–**3** in DMSO-*d*_6_; Figure S26: LC chromatograms of _L_ and _D_-FDLA derivatives of proline from spirotryprostatin H (**1**); Figure S27: LC chromatograms of _L_ and _D_-FDLA derivatives of proline from fumitremorgins O (**2**) and P (**3**); Figure S28: β-tubulin gene sequence of strain SCH3-Sd2.

## Abbreviations

The following abbreviations are used in this manuscript:

COSY	Correlated Spectroscopy
DKPs	Diketopiperazines
ECD	Electronic Circular Dichroism
HMBC	Heteronuclear Multiple Bond Correlation
HSQC	Heteronuclear Single-Quantum Correlation
HPLC	High-performance liquid chromatography
MIC	Minimum inhibitory concentration
NMR	Nuclear magnetic resonance
NOESY	Nuclear Overhauser Effect
OSMAC	One strain−many compounds
PDB	Potato Dextrose Broth
TFA	Trifluoroacetic acid

## References

[1] Schueffler A., Anke T. (2014). Fungal natural products in research and development. Nat. Prod. Rep..

[2] Eze P.M., Liu Y., Simons V.E., Ebada S.S., Kurtán T., Király S.B., Esimone C.O., Okoye F.B., Proksch P., Kalscheuer R. (2023). Two new metabolites from a marine-derived fungus *Penicillium ochrochloron*. Phytochem. Lett..

[3] Imhoff J.F. (2016). Natural products from marine fungi—Still an underrepresented resource. Mar. Drugs.

[4] Bugni T.S., Ireland C.M. (2004). Marine-derived fungi: A chemically and biologically diverse group of microorganisms. Nat. Prod. Rep..

[5] Yang X., Liu J., Mei J., Jiang R., Tu S., Deng H., Liu J., Yang S., Li J. (2021). Origins, structures, and bioactivities of secondary metabolites from marine-derived *Penicillium* fungi. Mini Rev. Med. Chem..

[6] Ma H.-G., Liu Q., Zhu G.-L., Liu H.-S., Zhu W.-M. (2016). Marine natural products sourced from marine-derived *Penicillium* fungi. J. Asian Nat. Prod. Res..

[7] Borthwick A.D. (2012). 2,5-Diketopiperazines: Synthesis, reactions, medicinal chemistry, and bioactive natural products. Chem. Rev..

[8] Zhang Y.-H., Du H.-F., Liu Y.-F., Cao F., Luo D.-Q., Wang C.-Y. (2024). Novel anti-inflammatory diketopiperazine alkaloids from the marine-derived fungus *Penicillium brasilianum*. Appl. Microbiol. Biotechnol..

[9] Reyes F., Bills G.F., Durán-Patrón R. (2022). Strategies for the discovery of fungal natural products. Front. Media SA.

[10] Romano S., Jackson S.A., Patry S., Dobson A.D. (2018). Extending the “one strain many compounds”(OSMAC) principle to marine microorganisms. Mar. Drugs.

[11] da Silva F.M.R., Paggi G.M., Brust F.R., Macedo A.J., Silva D.B. (2023). Metabolomic strategies to improve chemical information from OSMAC studies of endophytic fungi. Metabolites.

[12] Cui C.-B., Kakeya H., Osada H. (1996). Novel mammalian cell cycle inhibitors, spirotryprostatins A and B, produced by *Aspergillus fumigatus*, which inhibit mammalian cell cycle at G2/M phase. Tetrahedron.

[13] Liu J., Gao W.-J., Fang C.-H., Li Y., Liu X.-Y., Lv M.-J., Yue J.-M., Yu J.-H. (2025). Indole diketopiperazine alkaloids from a soil-derived fungus *Aspergillus* sp. KYS-11. Fitoterapia.

[14] Wang F., Fang Y., Zhu T., Zhang M., Lin A., Gu Q., Zhu W. (2008). Seven new prenylated indole diketopiperazine alkaloids from holothurian-derived fungus *Aspergillus fumigatus*. Tetrahedron.

[15] Yamazaki M., Fujimoto H., Kawasaki T. (1975). The structure of a tremorgenic metabolite from *Aspergillus fumigatus* fres., fumitremorgin a. Tetrahedron Lett..

[16] Peng J., Gao H., Li J., Ai J., Geng M., Zhang G., Zhu T., Gu Q., Li D. (2014). Prenylated indole diketopiperazines from the marine-derived fungus *Aspergillus versicolor*. J. Org. Chem..

[17] Fraley A.E., Sherman D.H. (2020). Enzyme evolution in fungal indole alkaloid biosynthesis. FEBS J..

[18] Borgman P., Lopez R.D., Lane A.L. (2019). The expanding spectrum of diketopiperazine natural product biosynthetic pathways containing cyclodipeptide synthases. Org. Biomol. Chem..

[19] Bhushan R., Brückner H. (2004). Marfey’s reagent for chiral amino acid analysis: A review. Amino Acids.

[20] Shi H., Jiang J., Zhang H., Jiang H., Su Z., Liu D., Jie L., He F. (2022). Antibacterial spirooxindole alkaloids from *Penicillium brefeldianum* inhibit dimorphism of pathogenic smut fungi. Front. Microbiol..

[21] Gao N., Shang Z.-C., Yu P., Luo J., Jian K.-L., Kong L.-Y., Yang M.-H. (2017). Alkaloids from the endophytic fungus *Penicillium brefeldianum* and their cytotoxic activities. Chin. Chem. Lett..

[22] Yang J., Lin D., Yang L., Li F., Yang Y., Cui X., Zhang R., Yang X. (2025). Four novel alkaloids from *Aspergillus fumigatus* and their antimicrobial activities. Fitoterapia.

[23] Zgurskaya H.I., López C.A., Gnanakaran S. (2015). Permeability barrier of Gram-negative cell envelopes and approaches to bypass it. ACS Infect. Dis..

[24] Štumpf S., Hostnik G., Primožič M., Leitgeb M., Salminen J.-P., Bren U. (2020). The effect of growth medium strength on minimum inhibitory concentrations of tannins and tannin extracts against *E. coli*. Molecules.

[25] Ramkissoon A., Seepersaud M., Maxwell A., Jayaraman J., Ramsubhag A. (2020). Isolation and antibacterial activity of indole alkaloids from *Pseudomonas aeruginosa* UWI-1. Molecules.

[26] Chung D., Baek K., Bae S.S., Jung J. (2019). Identification and characterization of a marine-derived chitinolytic fungus, *Acremonium* sp. YS2-2. J. Microbiol..

[27] Glass N.L., Donaldson G.C. (1995). Development of primer sets designed for use with the PCR to amplify conserved genes from filamentous ascomycetes. Appl. Environ. Microbiol..

[28] Nam S.-J., Kauffman C.A., Jensen P.R., Fenical W. (2011). Isolation and characterization of actinoramides A–C, highly modified peptides from a marine *Streptomyces* sp.. Tetrahedron.

