# Supplementary material for: Isolation of Three New Diketopiperazine Alkaloids from Penicillium sp. SCH3-Sd2
Source: Molecules. 2026 Jan 1;31(1):149. doi: 10.3390/molecules31010149 (PMC12786964; doi:10.3390/molecules31010149)
Supplement: Supplementary file 1 [file molecules-31-00149-s001.zip › molecules-4038168-supplementary.pdf]

Supplementary Information

Isolation of Three New Diketopiperazine Alkaloids from *Penicillium* sp. SCH3-Sd2

Eun-Young Lee [1,†], Qian Gao [2,†], Hui Tan [2], Prima F. Hillman [3], Dawoon Chung [4], Grace Choi [5], Hiyoung Kim [6], Kyung-Min Lim [1,*] and Sang-Jip Nam [2,7,*]

[1] College of Pharmacy, Ewha Womans University, Seoul 03760, Republic of Korea; younglee0124@naver.com (E.-Y.L.); kmlim@ewha.ac.kr (K.-M.L.)
[2] Department of Chemistry and Nanoscience, Ewha Womans University, Seoul 03760, Republic of Korea; sjnam@ewha.ac.kr (S.-J.N.)
[3] Department of Chemistry, Faculty of Mathematics and Natural Sciences, Universitas Andalas, Kampus Limau Manis, Padang 25163, Indonesia; prima.fitria@sci.unand.ac.id (P.H.)
[4] Department of Biomaterial Research, National Marine Biodiversity Institute of Korea, Seocheon 33662, Republic of Korea; dwchung@mabik.re.kr (D.C.)
[5] Department of Marine Ecological Biotechnology, Kunsan National University, Gunsan 54150, Republic of Korea; gchoi@kunsan.ac.kr (G.C.)
[6] Department of Biomedical Science and Engineering, Konkuk University, Seoul 05029, Republic of Korea; reihyoung@konkuk.ac.kr (H.K.)
[7] Graduate Program in Innovative Biomaterials Convergence, Ewha Womans University, Seoul 03760, Republic of Korea; sjnam@ewha.ac.kr (S.-J.N.)
* Correspondence: sjnam@ewha.ac.kr (S.-J.N.), Tel.: +82-2-3277-6805; kmlim@ewha.ac.kr (K.-M.L.), Tel.: +82-2-3277-3055

first authorship.

† These authors contributed equally to this work.

Table of Contents

Figure S1. ^{1}H NMR spectrum of spirotryprostatin H (**1**) in DMSO-d_6 ········ **S6**

Figure S2. ^{13}C NMR spectrum of spirotryprostatin H (**1**) in DMSO-d_6 ········ **S7**

Figure S3. COSY NMR spectrum of spirotryprostatin H (**1**) in DMSO-d_6 ········ **S8**

Figure S4. HSQC NMR spectrum of spirotryprostatin H (**1**) in DMSO-d_6 ········ **S9**

Figure S5. HMBC NMR spectrum of spirotryprostatin H (**1**) in DMSO-d_6 ········ **S10**

Figure S6. NOESY NMR spectrum of spirotryprostatin H (**1**) in DMSO-d_6 ········ **S11**

Figure S7. High resolution mass data of spirotryprostatin H (**1**) ········ **S12**

Figure S8. FT-IR spectrum of spirotryprostatin H (**1**) ········ **S13**

Figure S9. ^{1}H NMR spectrum of fumitremorgin O (**2**) in DMSO-d_6 ········ **S14**

Figure S10. ^{13}C NMR spectrum of fumitremorgin O (**2**) in DMSO-d_6 ········ **S15**

Figure S11. COSY NMR spectrum of fumitremorgin O (**2**) in DMSO-d_6 ········ **S16**

Figure S12. HSQC NMR spectrum of fumitremorgin O (**2**) in DMSO-d_6 ········ **S17**

Figure S13. HMBC NMR spectrum of fumitremorgin O (**2**) in DMSO-d_6 ········ **S18**

Figure S14. NOESY NMR spectrum of fumitremorgin O (**2**) in DMSO-*d*6 **S19**

Figure S15. High resolution mass data of fumitremorgin O (**2**) **S20**

Figure S16. FT-IR spectrum of fumitremorgin O (**2**) **S21**

Figure S17. ^{1}H NMR spectrum of fumitremorgin P (**3**) in DMSO-*d*6 **S22**

Figure S18. ^{13}C NMR spectrum of fumitremorgin P (**3**) in DMSO-*d*6 **S23**

Figure S19. COSY NMR spectrum of fumitremorgin P (**3**) in DMSO-*d*6 **S24**

Figure S20. HSQC NMR spectrum of fumitremorgin P (**3**) in DMSO-*d*6 **S25**

Figure S21. HMBC NMR spectrum of fumitremorgin P (**3**) in DMSO-*d*6 **S26**

Figure S22. NOESY NMR spectrum of fumitremorgin P (**3**) in DMSO-*d*6 **S27**

Figure S23. High resolution mass data of fumitremorgin P (**3**) **S28**

Figure S24. FT-IR spectrum of fumitremorgin P (**3**) **S29**

Figure S25. FT-IR spectrum of $CHCl_3$ background signal **S30**

Table S1. ECD data of compounds **1–3** **S31**

Table S2. NMR correlation data for compounds **1–3** in DMSO-*d*6 **S49**

Figure S26. LC chromatograms of L and D-FDLA derivatives of proline from spirotryprostatin H (**1**) ······ **S51**

Figure S27. LC chromatograms of L and D-FDLA derivatives of proline from fumitremorgins O (**2**) and P (**3**) ······ **S52**

Figure S28. β-tubulin gene sequence of strain SCH3-Sd2 ······ **S53**

Figure S1. ^{1}H NMR spectrum of spirotryprostatin H (**1**) in DMSO-d_6

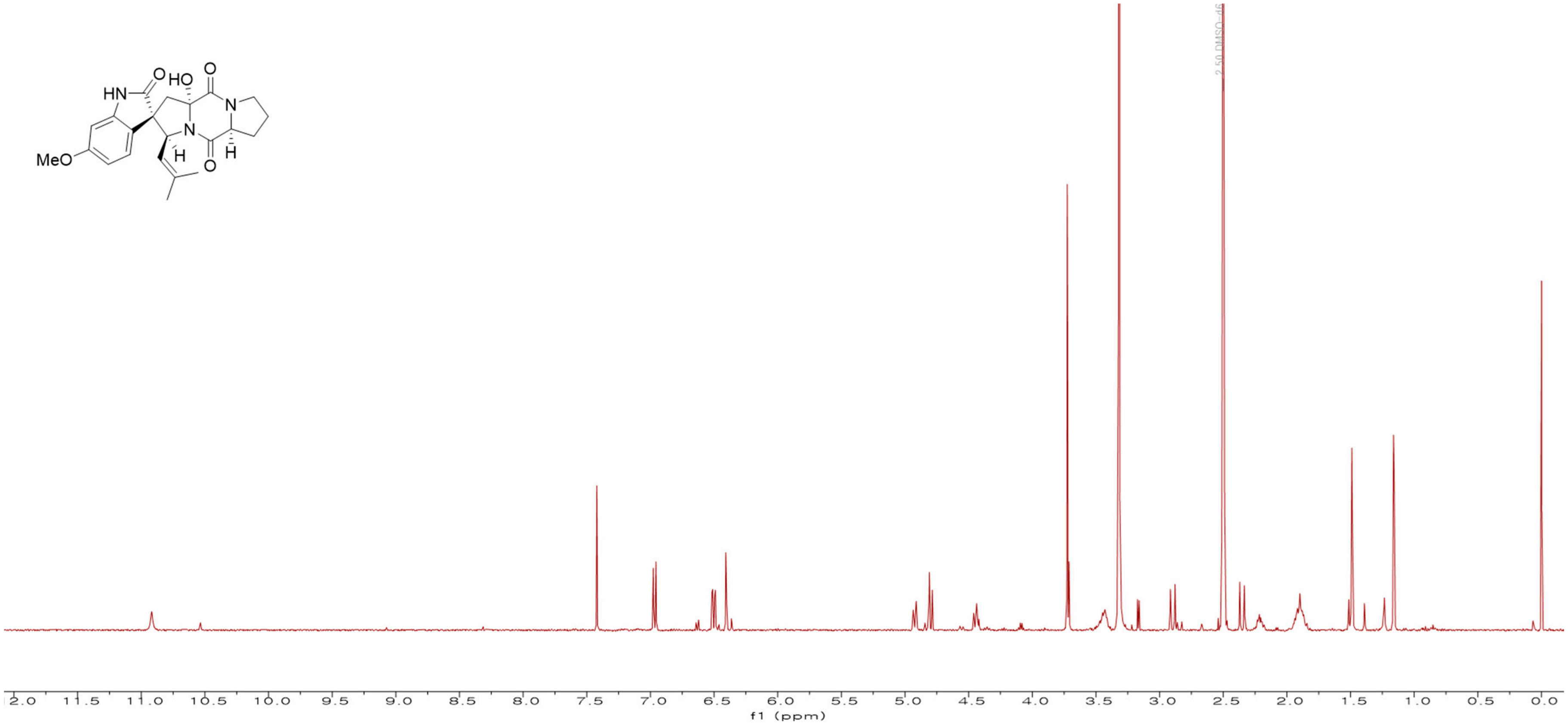

Figure S2. ^{13}C NMR spectrum of spirotryprostatin H (**1**) in DMSO-d_6

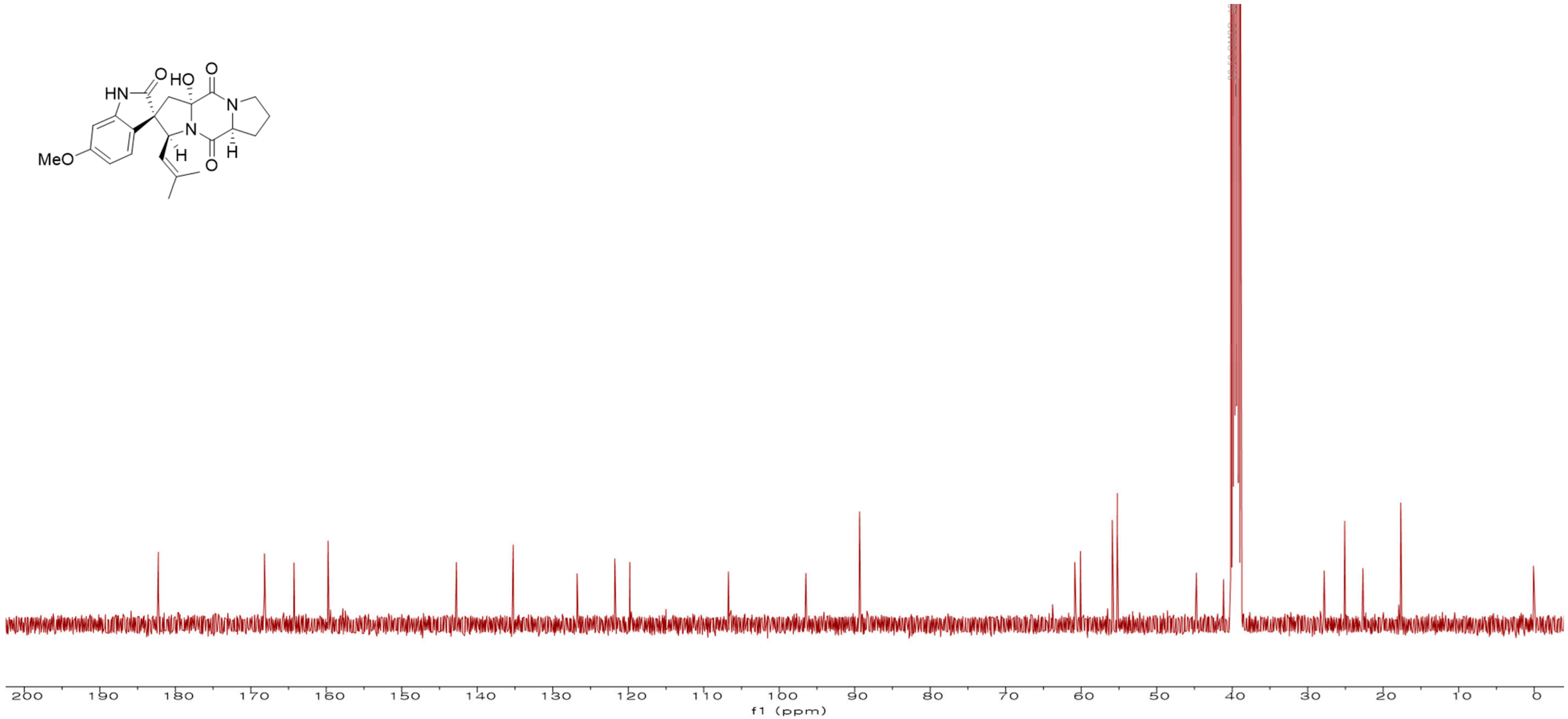

Figure S3. COSY NMR spectrum of spirotryprostatin H (**1**) in DMSO-*d*₆

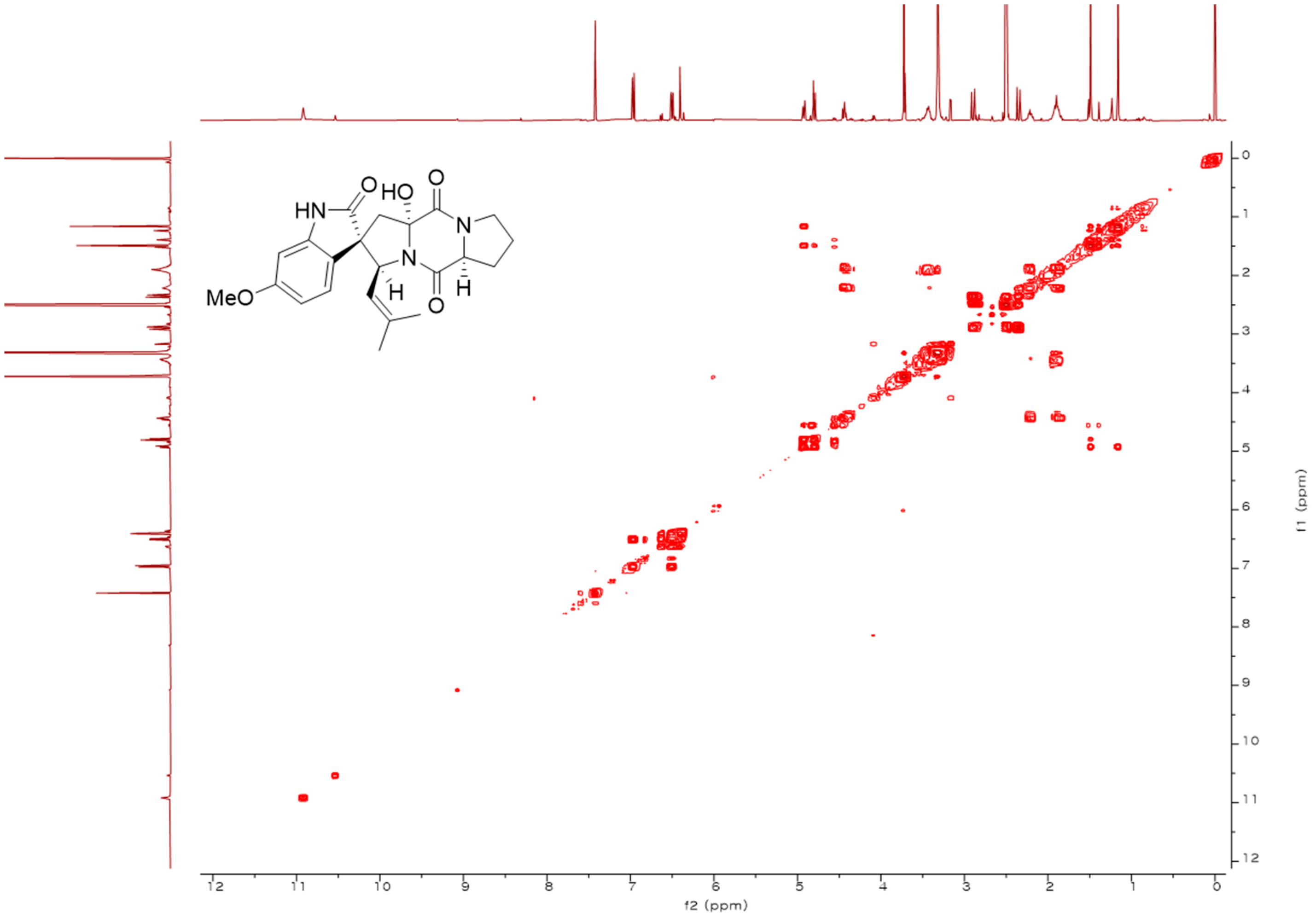

Figure S4. HSQC NMR spectrum of spirotryprostatin H (**1**) in DMSO-d_6

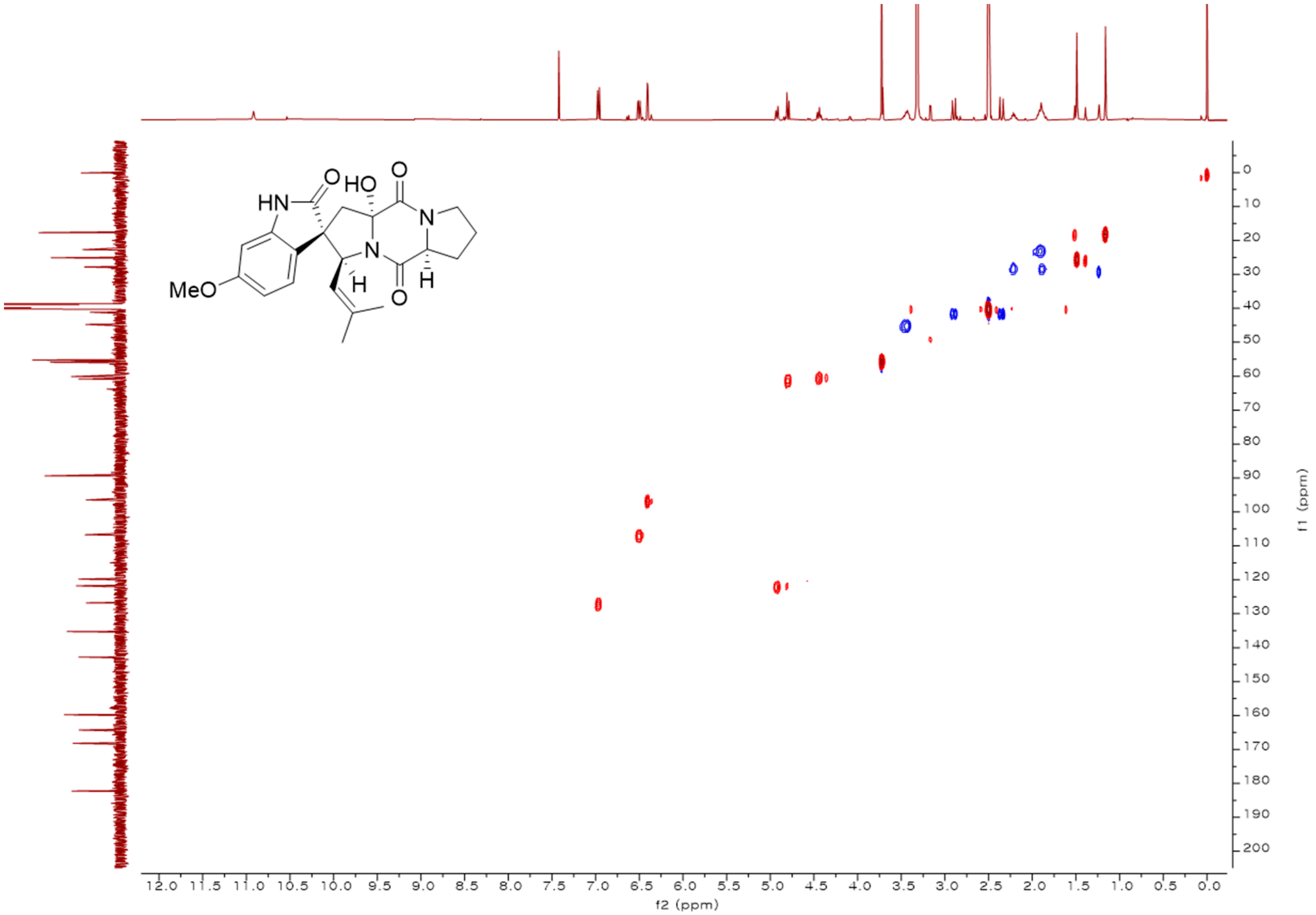

Figure S5. HMBC NMR spectrum of spirotryprostatin H (**1**) in DMSO-d_6

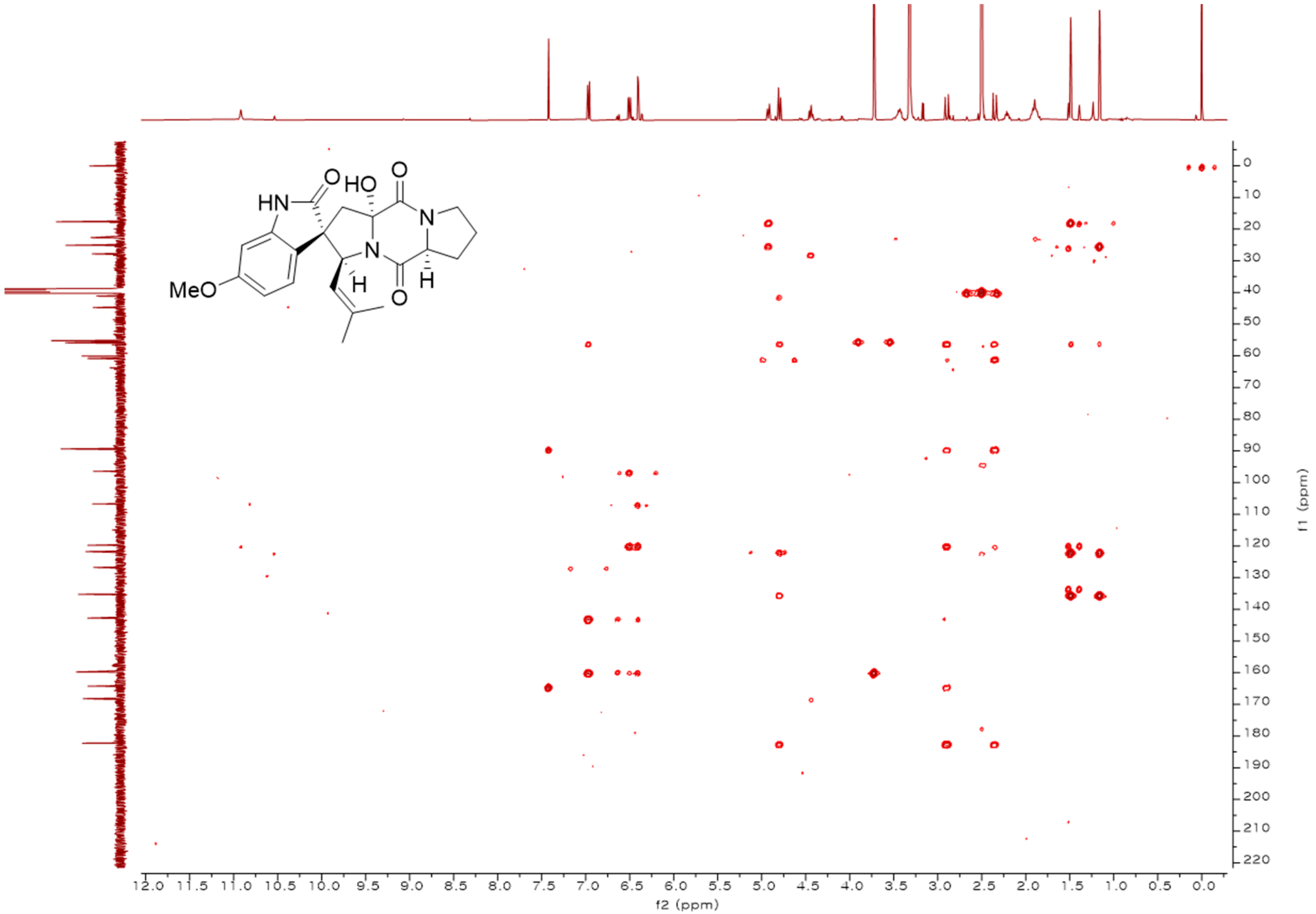

Figure S6. NOESY NMR spectrum of spirotryprostatin H (**1**) in DMSO-*d*

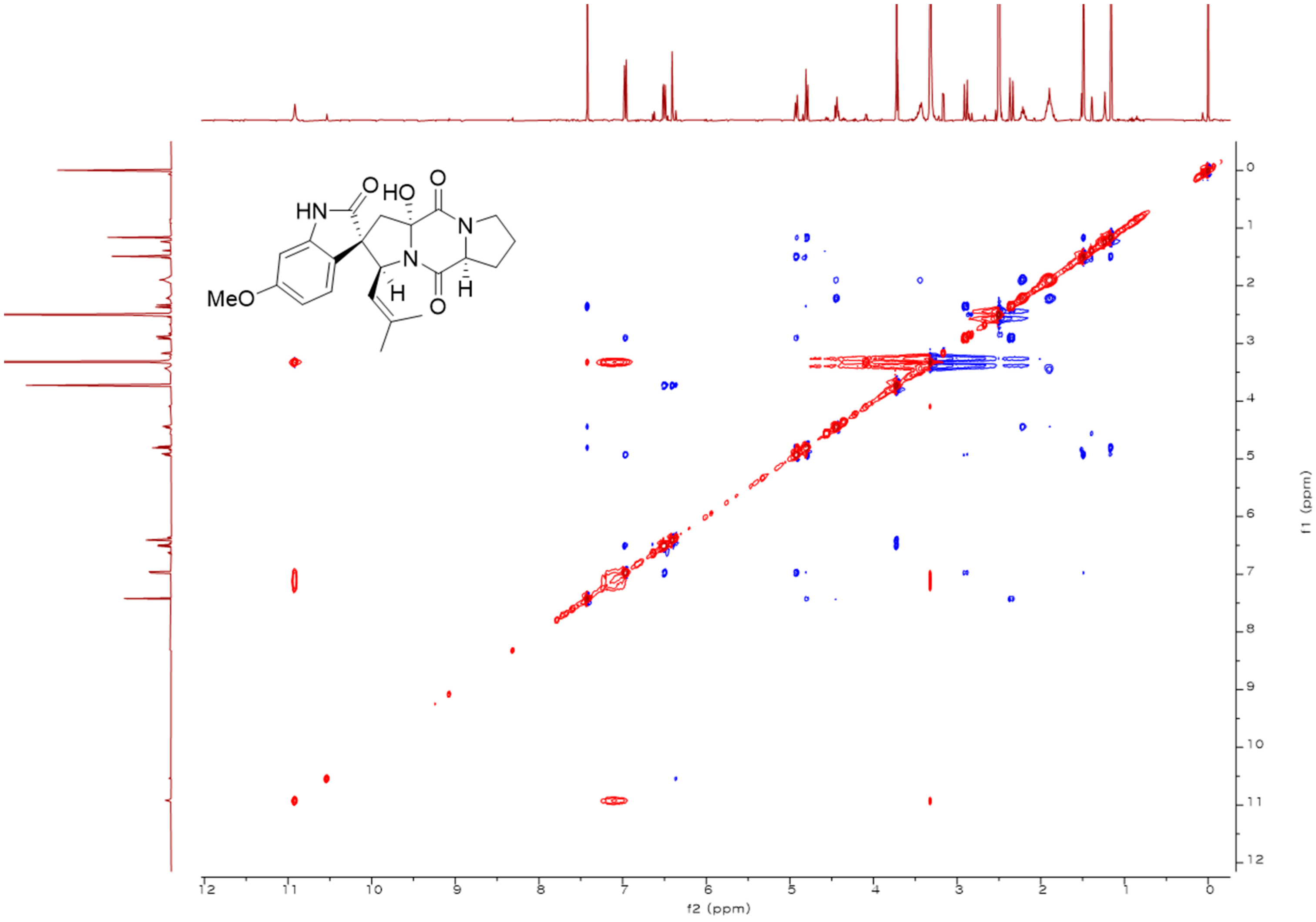

Figure S7. High resolution mass data of spirotryprostatin H (**1**)

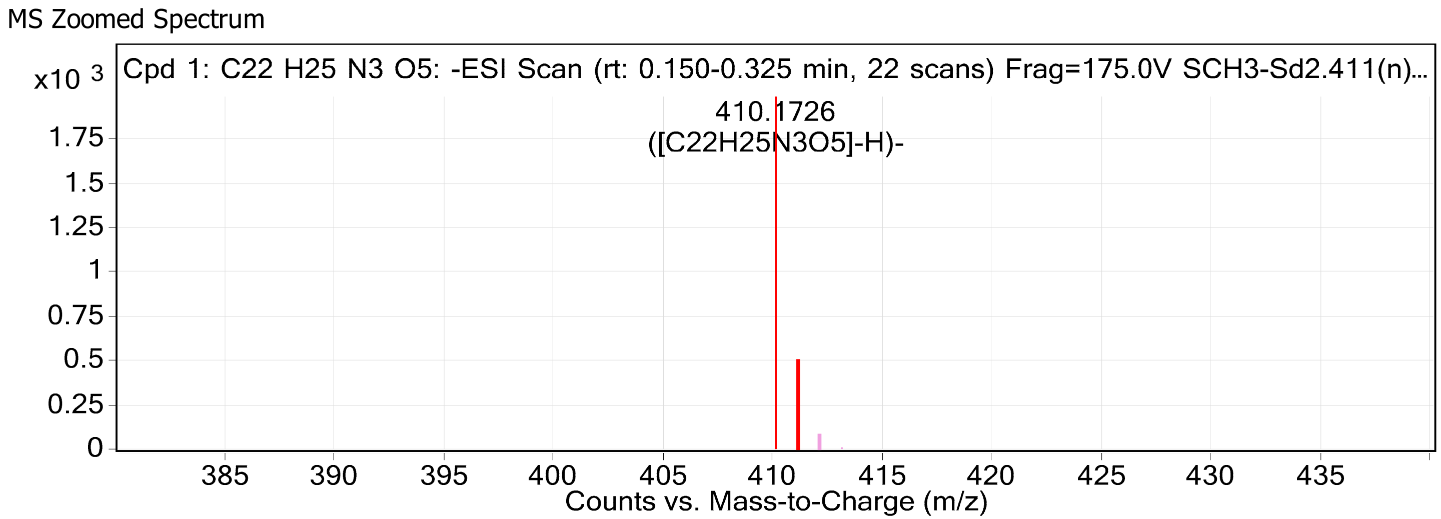

MS Spectrum Peak List

m/z	*Calc m/z*	Diff(ppm)	z	Abund	Formula	Ion
410.1726	410.1721	-1	1	1988.75	C22H25N3O5	(M-H)-
411.1755	411.1753	-0.5	1	422.49	C22H25N3O5	(M-H)-

Figure S8. FT-IR spectrum of spirotryprostatin H (**1**)

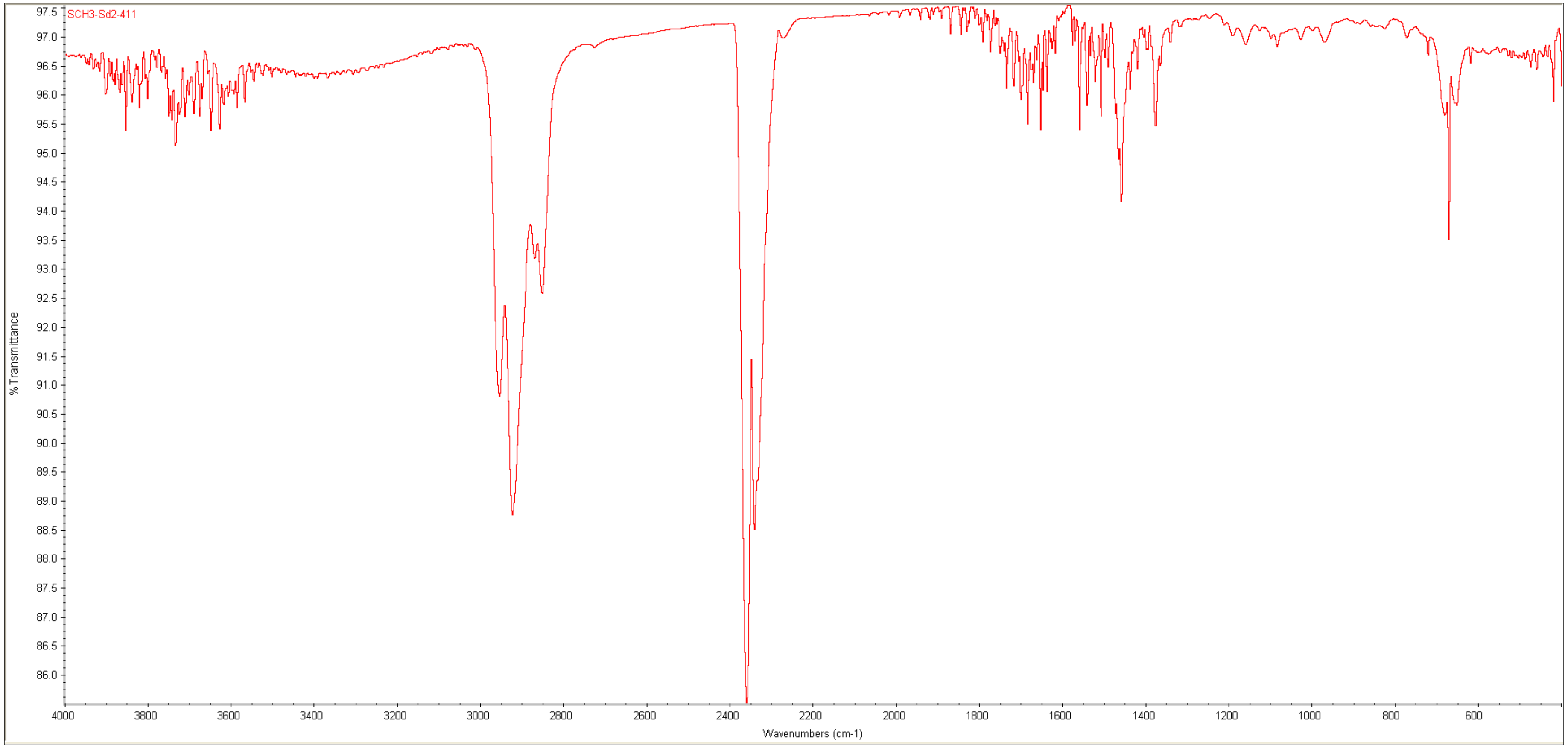

Figure S9. ^{1}H NMR spectrum of fumitremorgin O (**2**) in DMSO-d_6

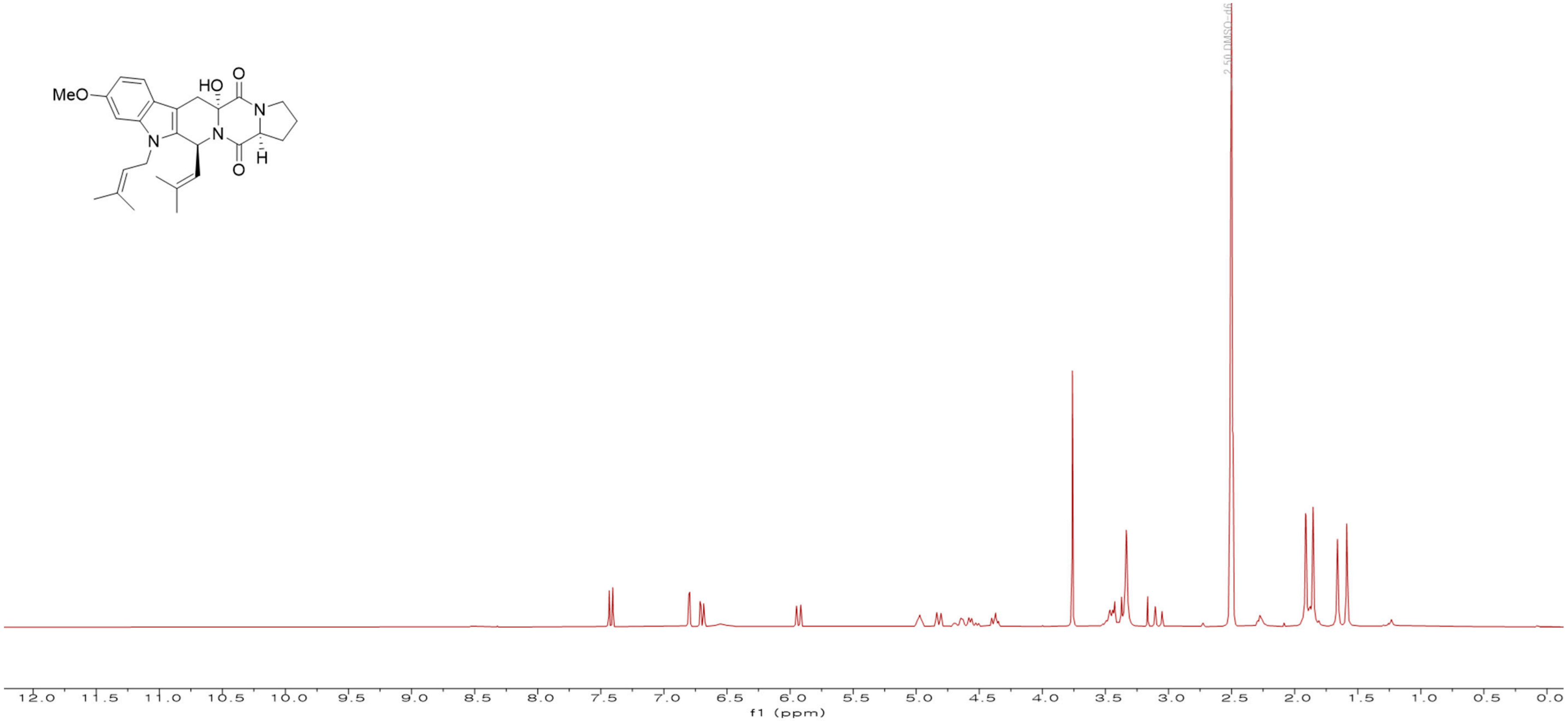

Figure S10. ^{13}C NMR spectrum of fumitremorgin O (**2**) in DMSO-d_6

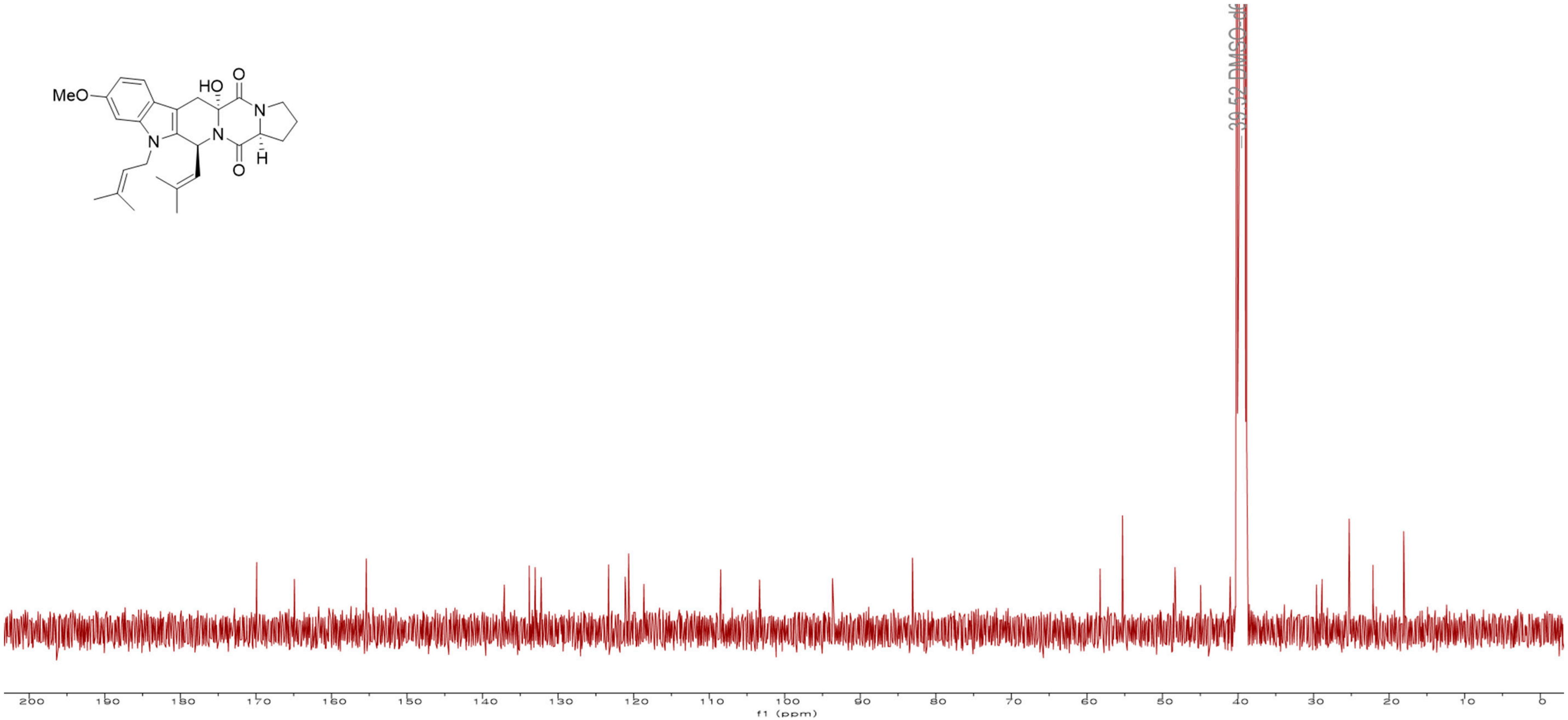

Figure S11. COSY NMR spectrum of fumitremorgin O (**2**) in DMSO-d_6

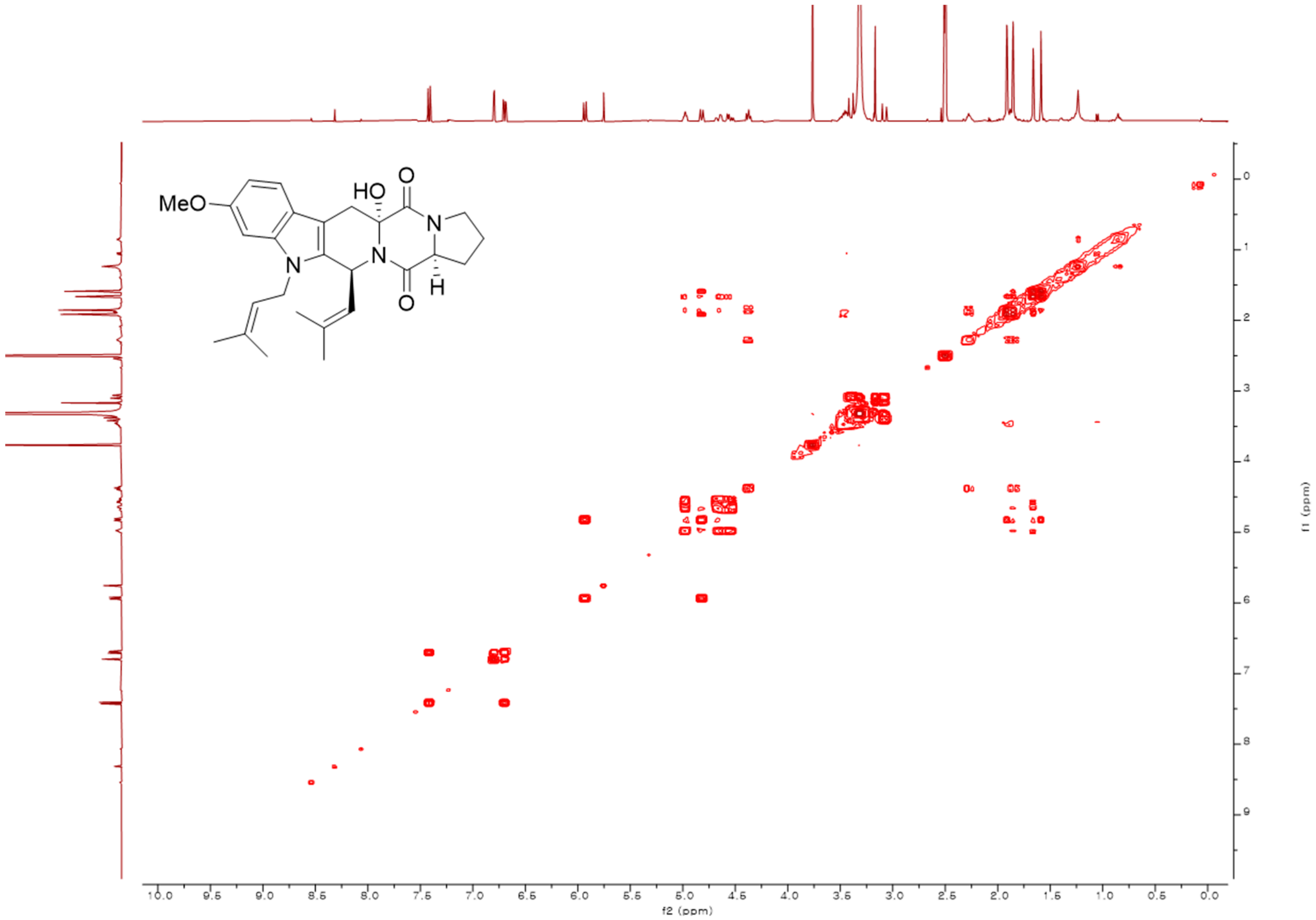

Figure S12. HSQC NMR spectrum of fumitremorgin O (**2**) in DMSO-d_6

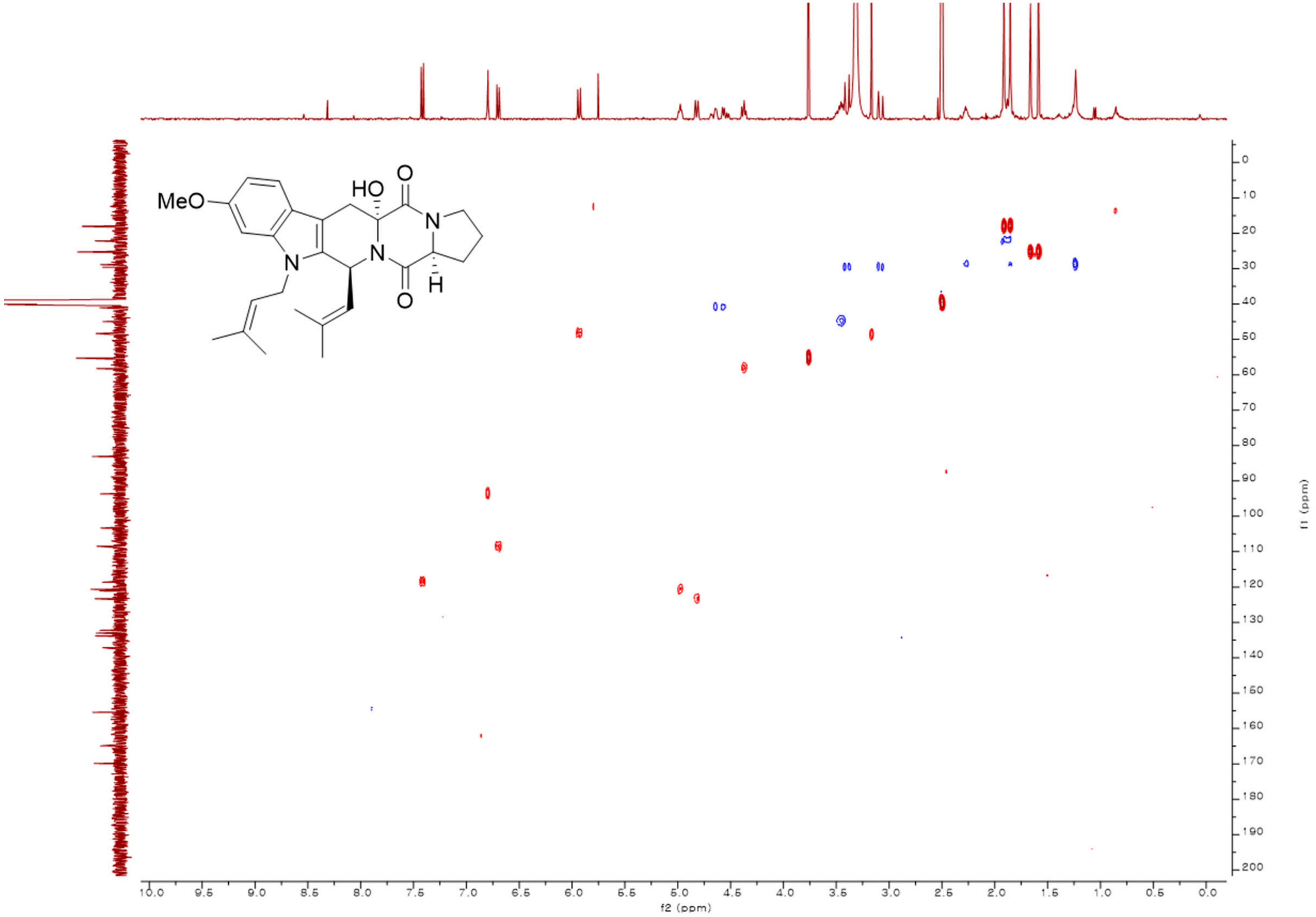

Figure S13. HMBC NMR spectrum of fumitremorgin O (**2**) in DMSO-d_6

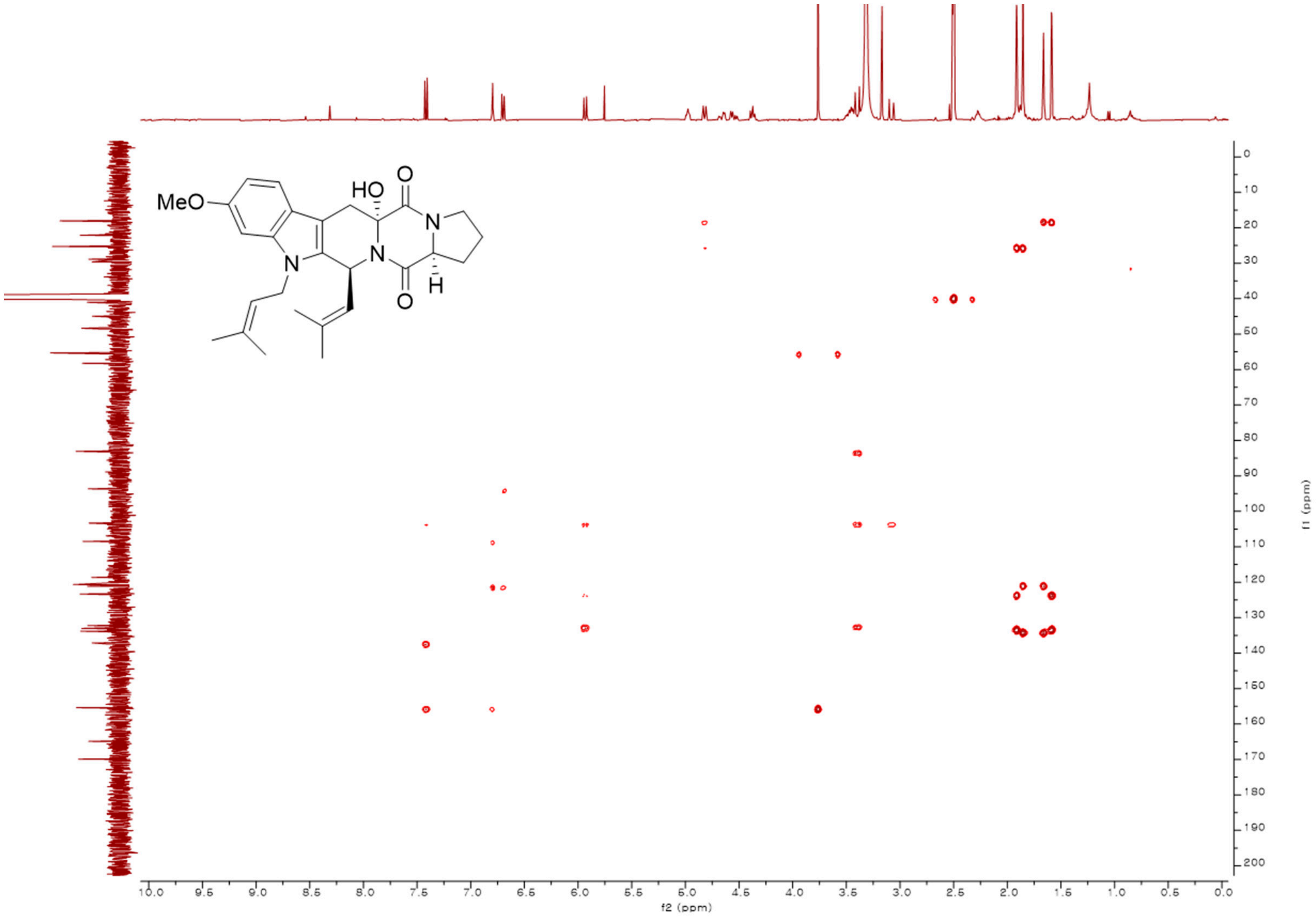

Figure S14. NOESY NMR spectrum of fumitremorgin O (**2**) in DMSO-d_6

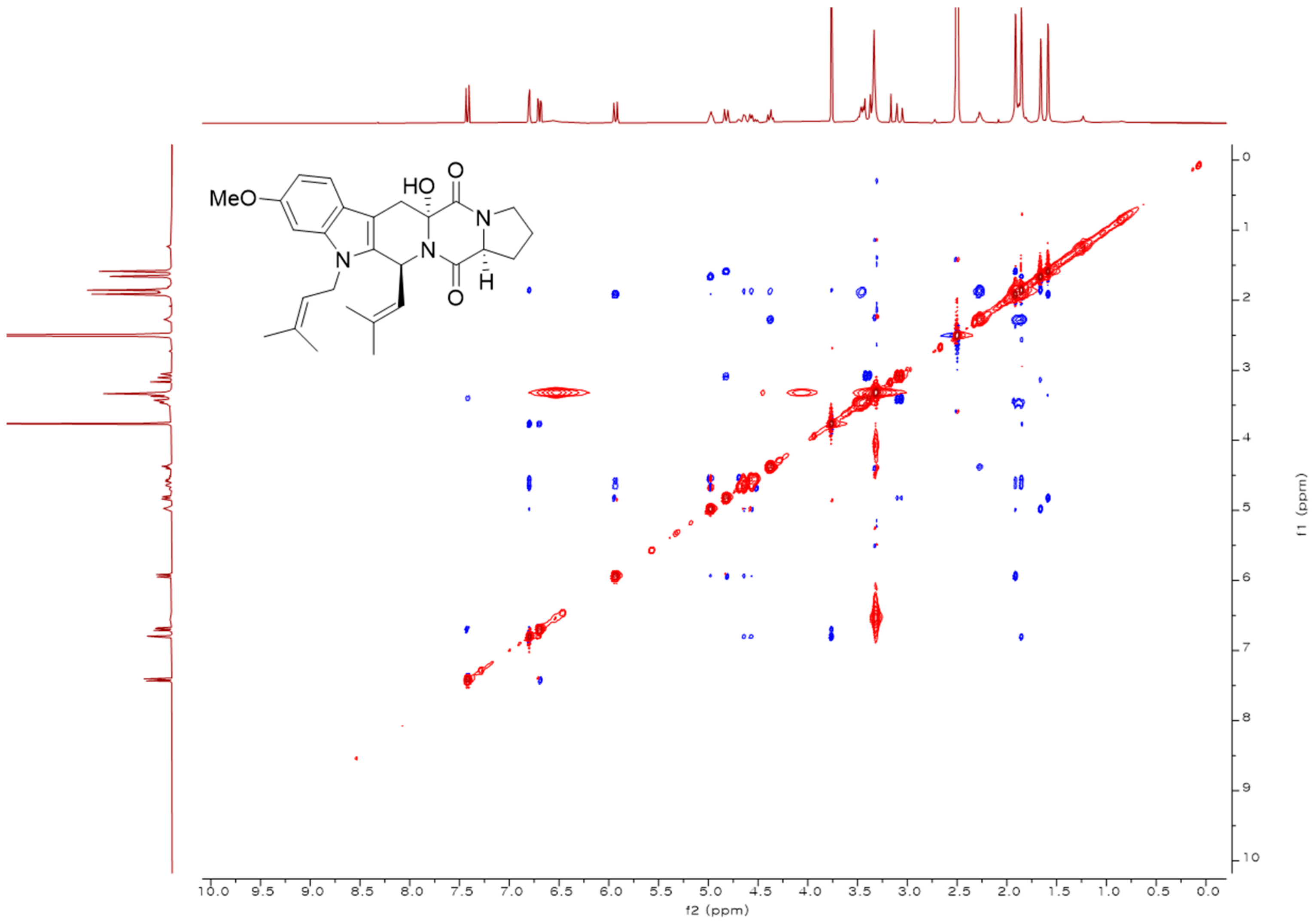

Figure S15. High resolution mass data of fumitremorgin O (**2**)

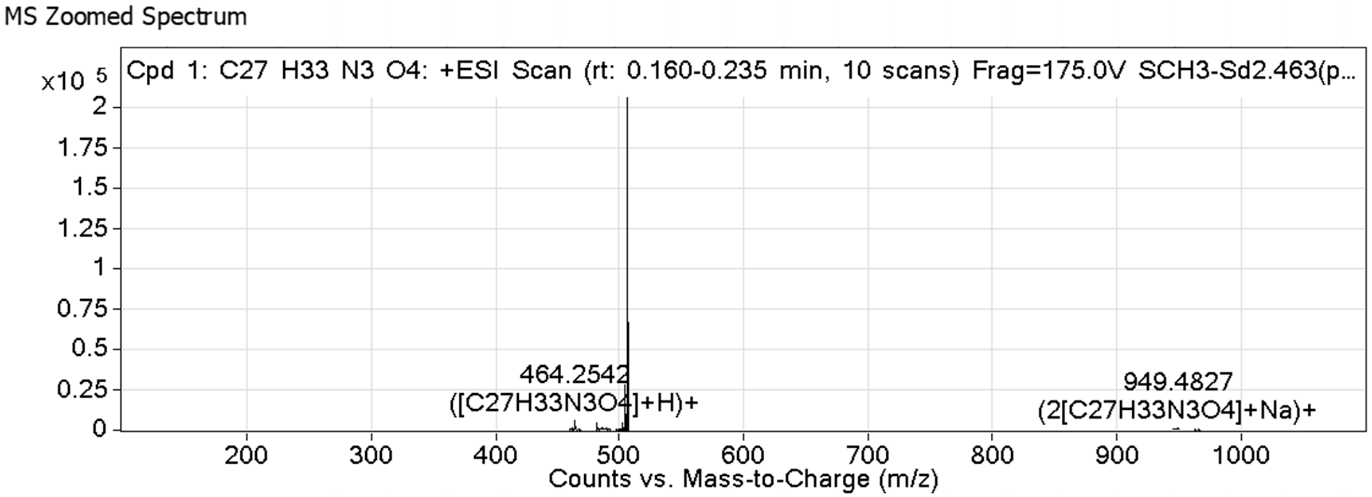

MS Spectrum Peak List

m/z	*Calc m/z*	Diff(ppm)	z	Abund	Formula	Ion
464.2542	464.2544	0.46	1	5963.06	C27H33N3O4	(M+H)+
465.2582	465.2576	-1.31	1	1892.22	C27H33N3O4	(M+H)+
466.2624	466.2604	-4.28	1	380.69	C27H33N3O4	(M+H)+
486.2361	486.2363	0.44	1	1538.15	C27H33N3O4	(M+Na)+
487.2414	487.2395	-3.89	1	459.56	C27H33N3O4	(M+Na)+
502.224	502.2103	-27.41	1	595.86	C27H33N3O4	(M+K)+
949.4827	949.4834	0.73	1	1381.78	C27H33N3O4	(2M+Na)+
950.4856	950.4866	1.02	1	871.3	C27H33N3O4	(2M+Na)+
951.4935	951.4896	-4.13	1	309.82	C27H33N3O4	(2M+Na)+
965.4728	965.4574	-15.97	1	115.42	C27H33N3O4	(2M+K)+

Figure S16. FT-IR spectrum of fumitremorgin O (**2**)

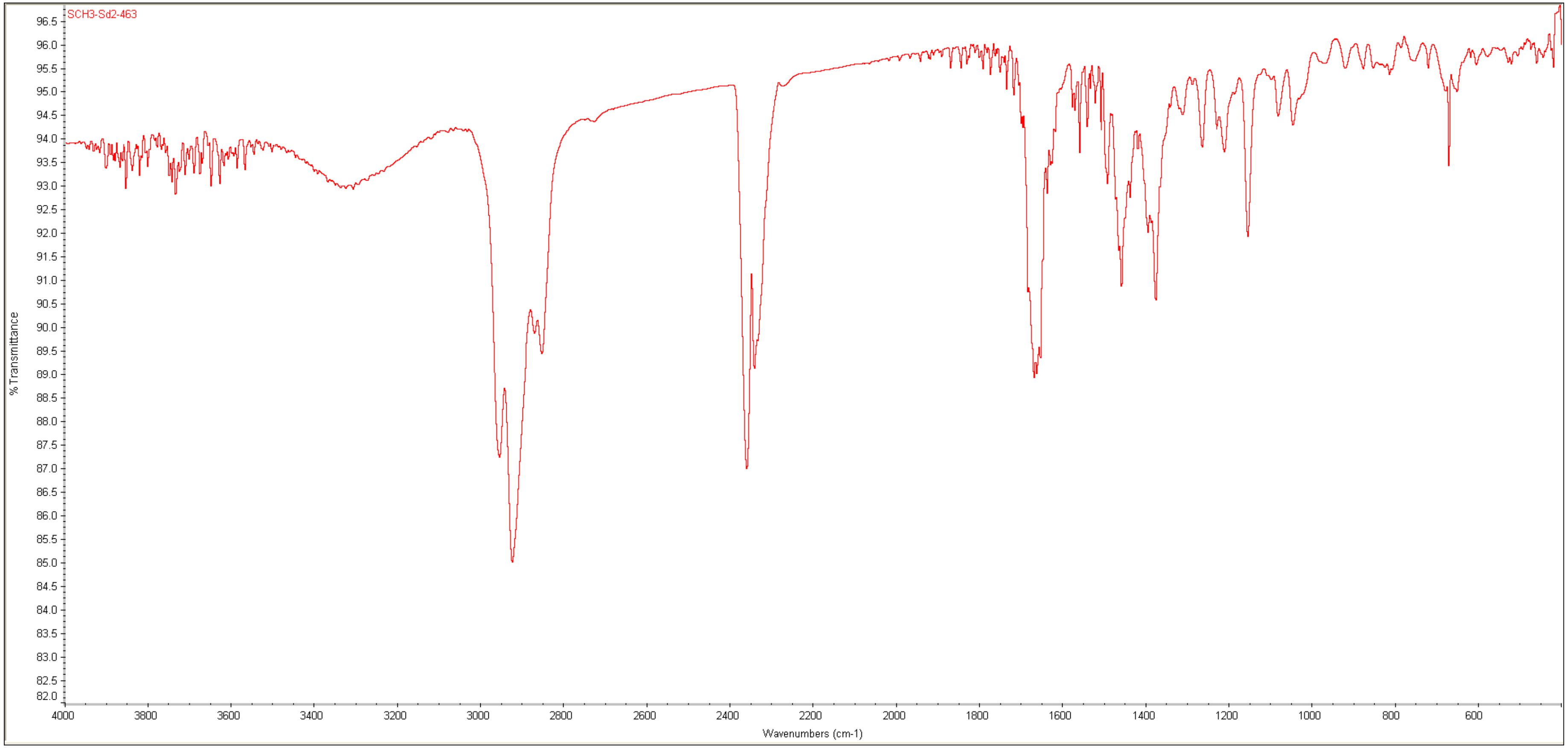

Figure S17. ^{1}H NMR spectrum of fumitremorgin P (**3**) in DMSO-d_6

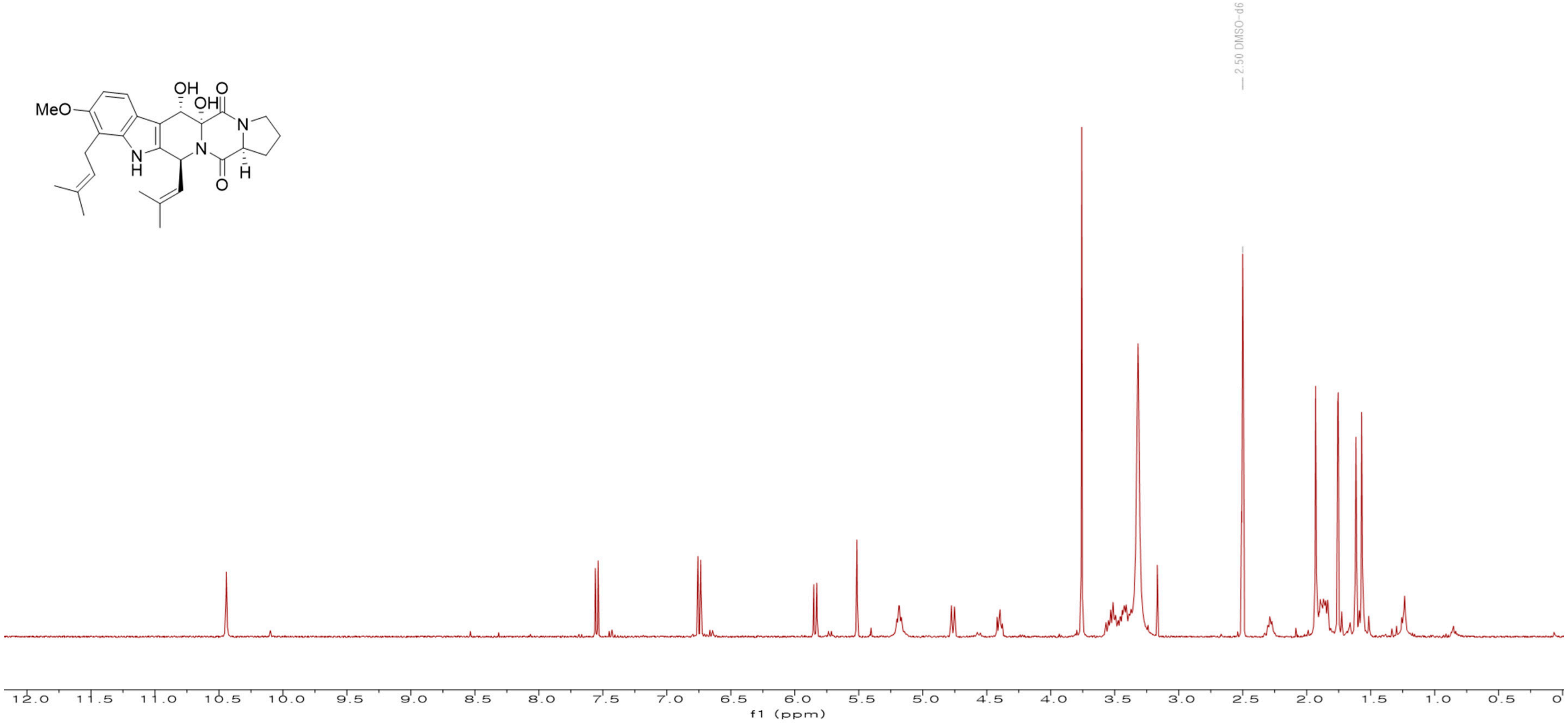

Figure S18. ^{13}C NMR spectrum of fumitremorgin P (**3**) in DMSO-d_6

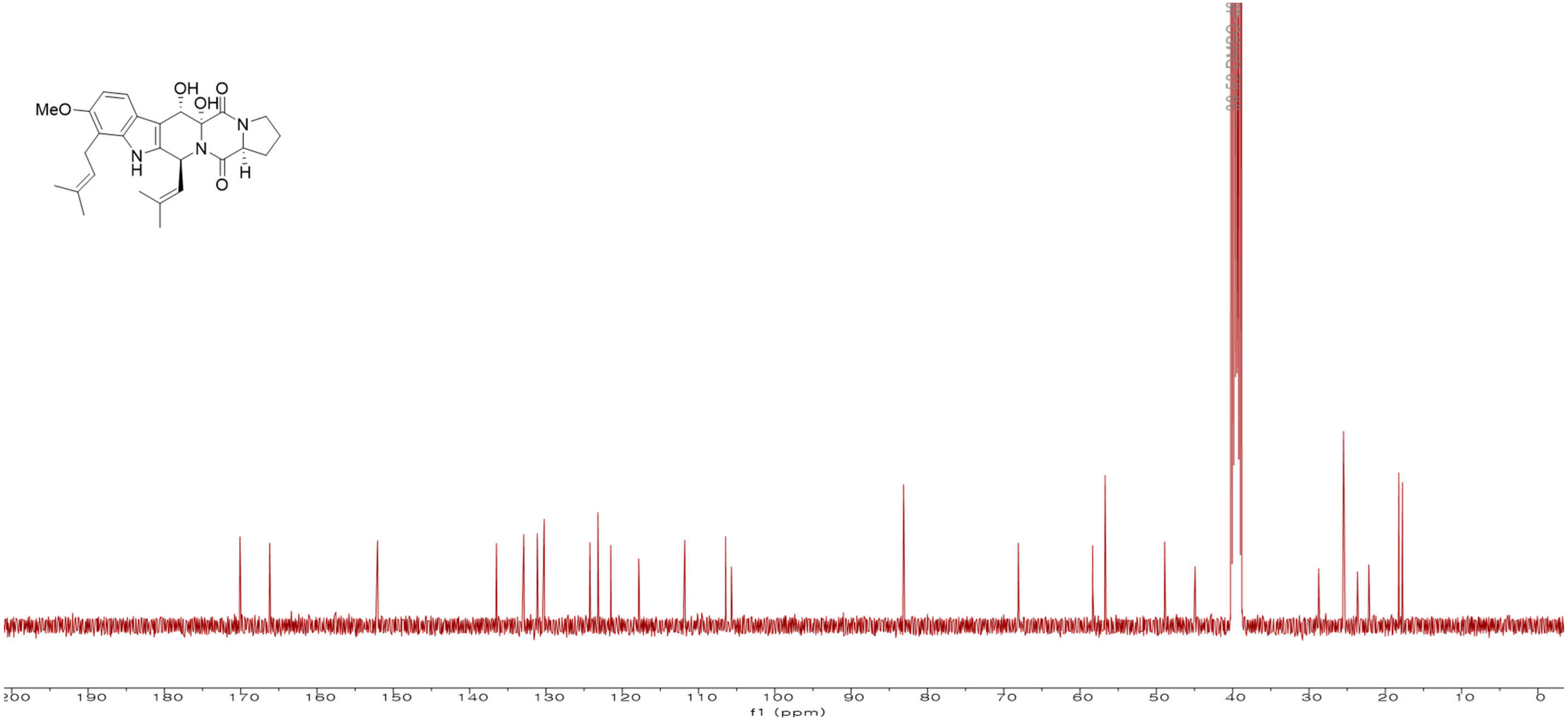

Figure S19. COSY NMR spectrum of fumitremorgin P (**3**) in DMSO-d_6

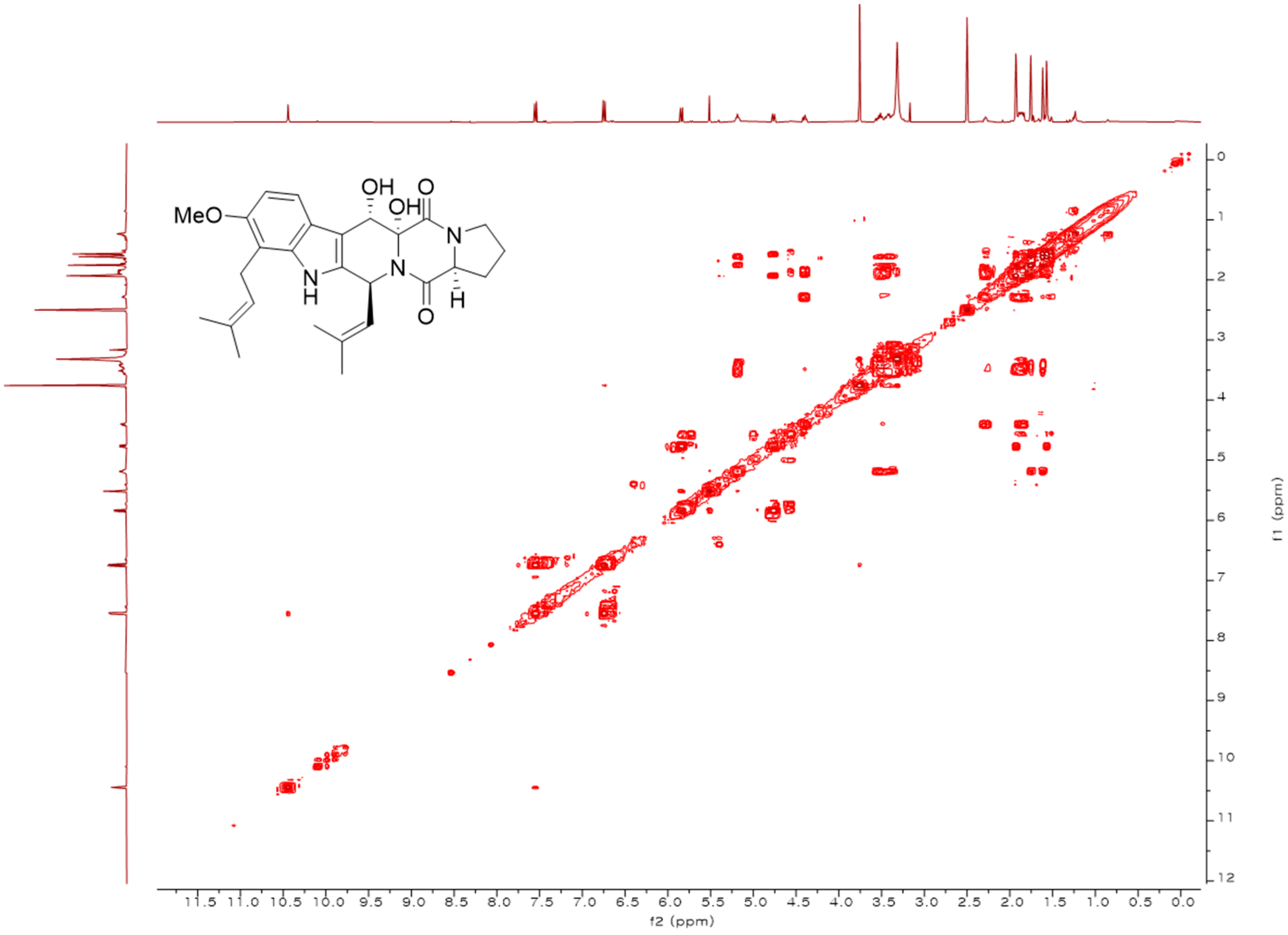

Figure S20. HSQC NMR spectrum of fumitremorgin P (**3**) in DMSO-d_6

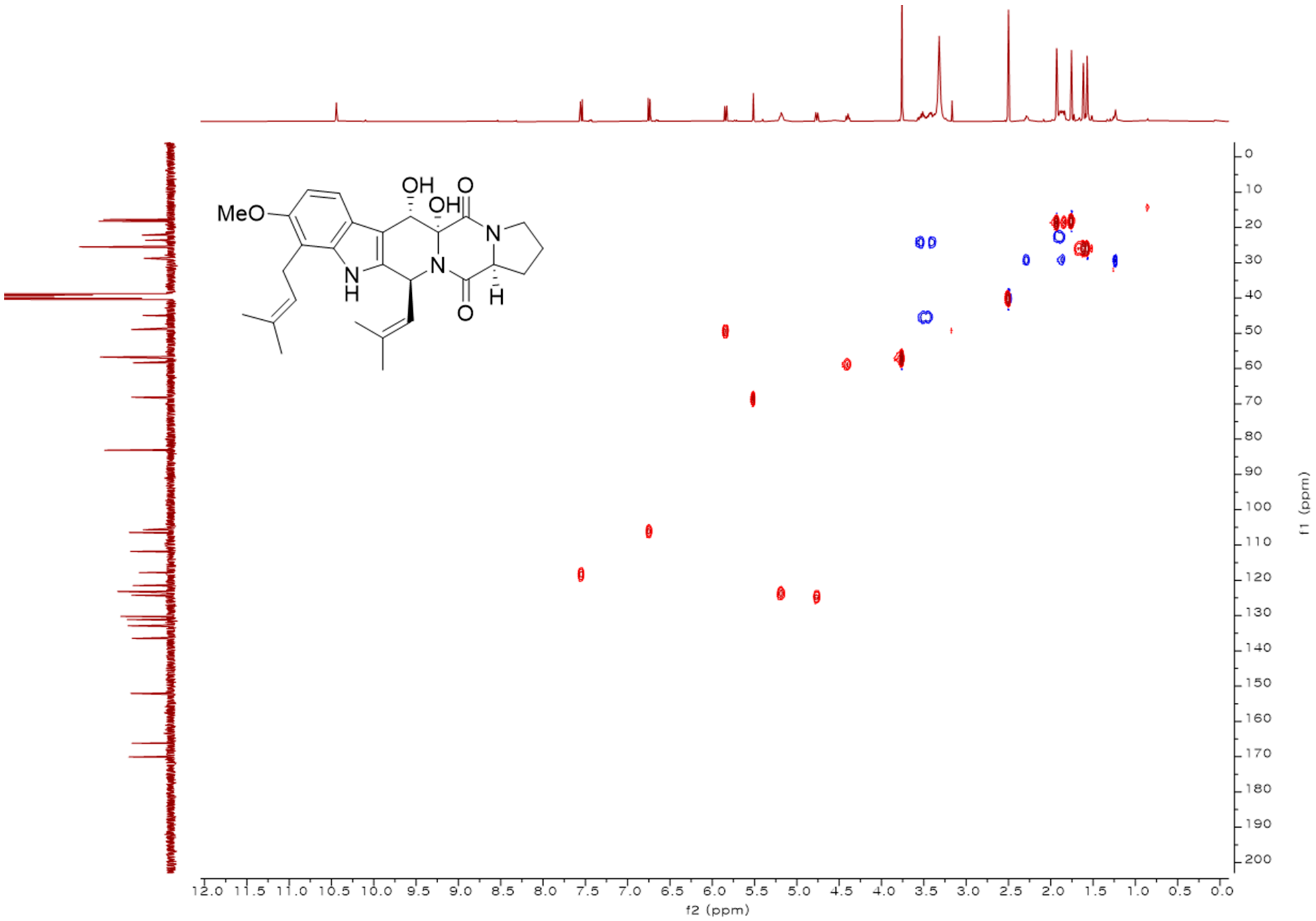

Figure S21. HMBC NMR spectrum of fumitremorgin P (**3**) in DMSO-d_6

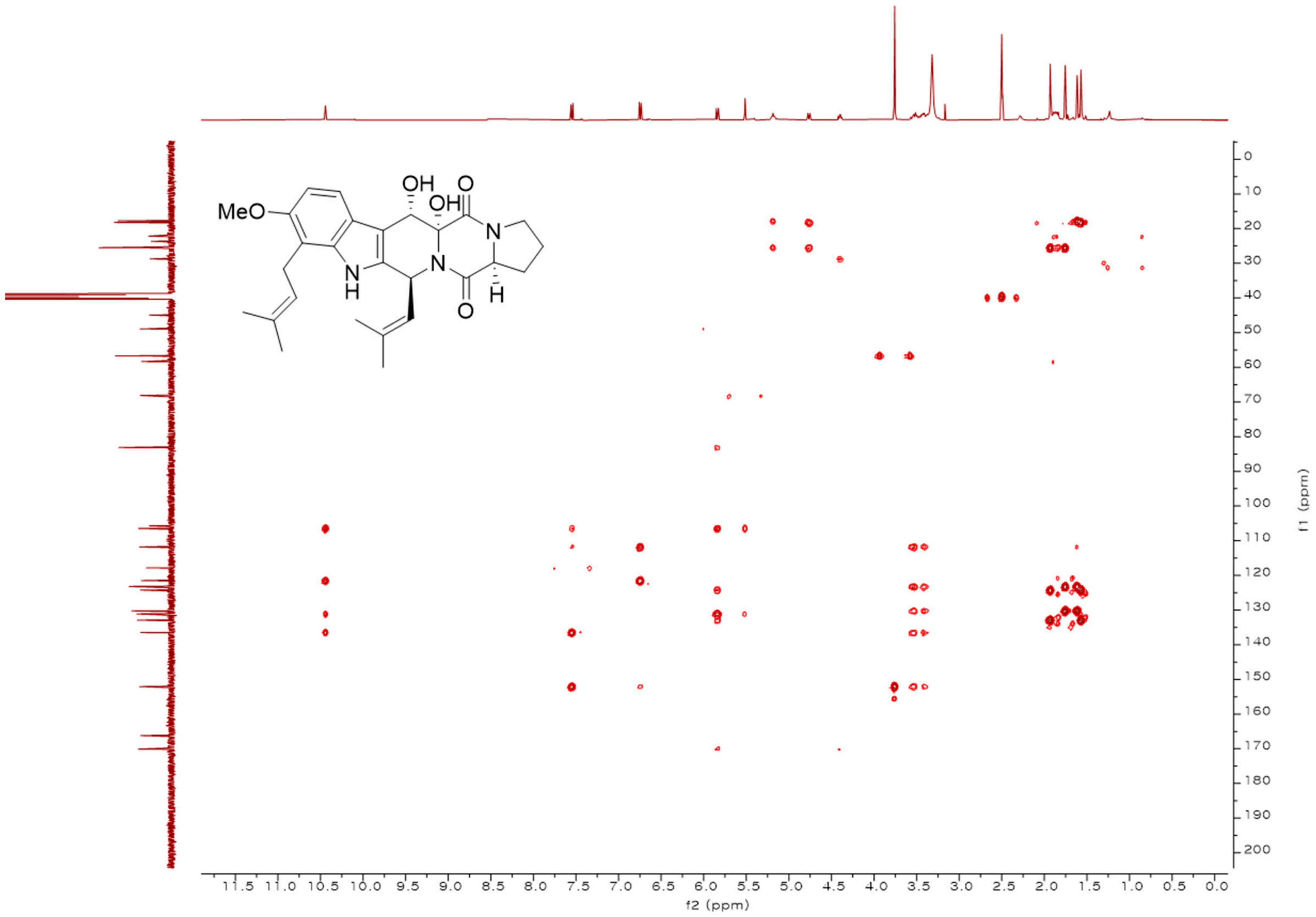

Figure S22. NOESY NMR spectrum of fumitremorgin P (**3**) in DMSO-*d*6

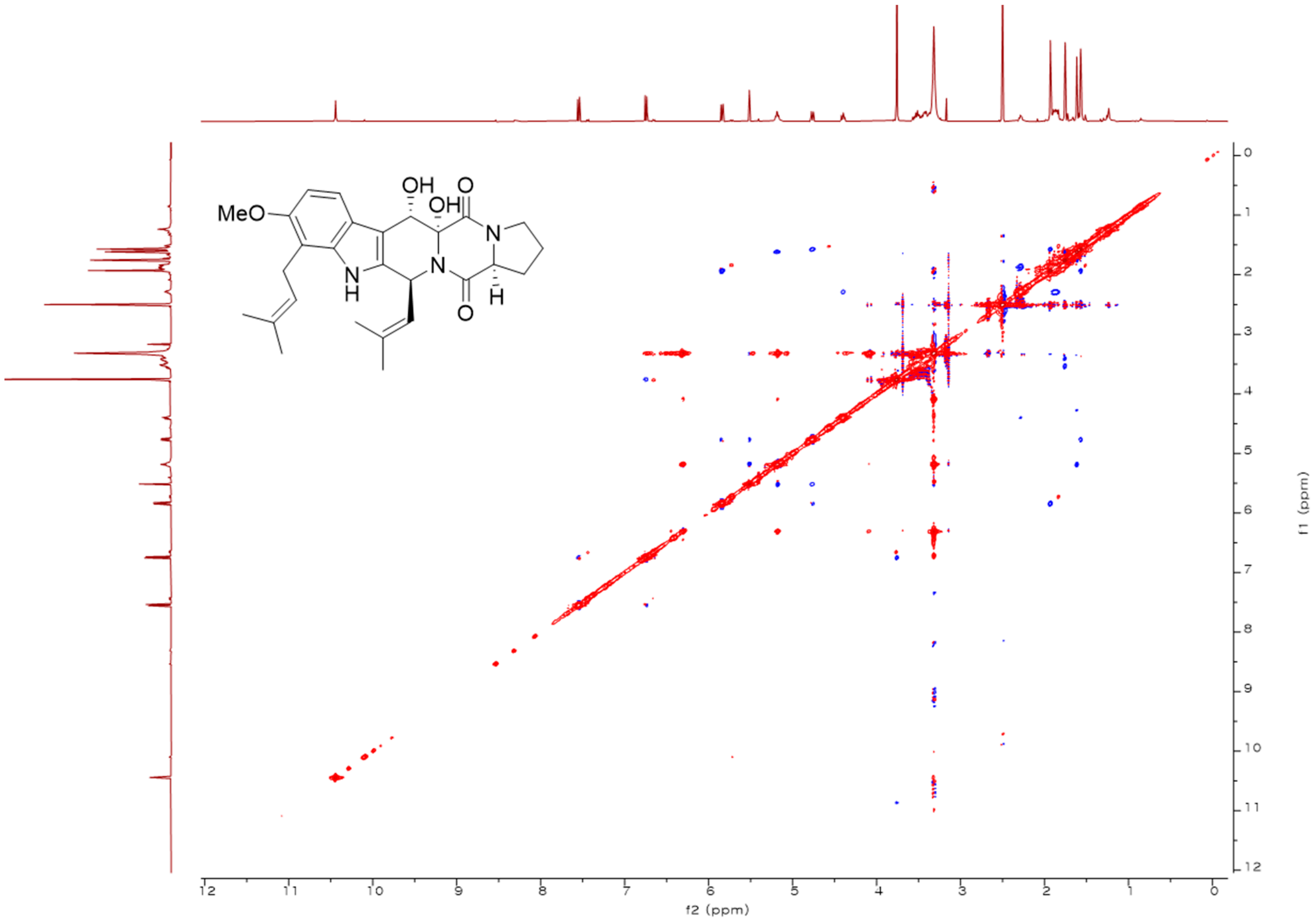

Figure S23. High resolution mass data of fumitremorgin P (**3**)

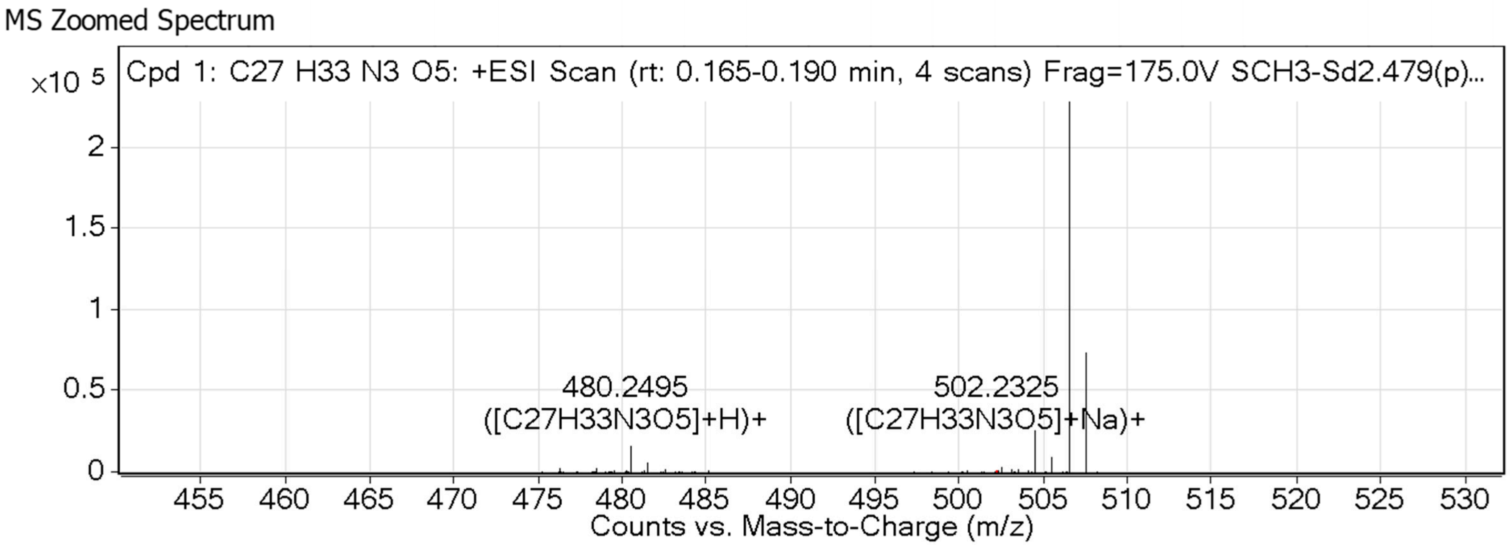

MS Spectrum Peak List

m/z	*Calc m/z*	Diff(ppm)	z	Abund	Formula	Ion
480.2495	480.2493	-0.44	1	502.71	C27H33N3O5	(M+H)+
502.2325	502.2312	-2.55	1	783.19	C27H33N3O5	(M+Na)+
503.2352	503.2344	-1.65	1	243.38	C27H33N3O5	(M+Na)+

Figure S24. FT-IR spectrum of fumitremorgin P (**3**)

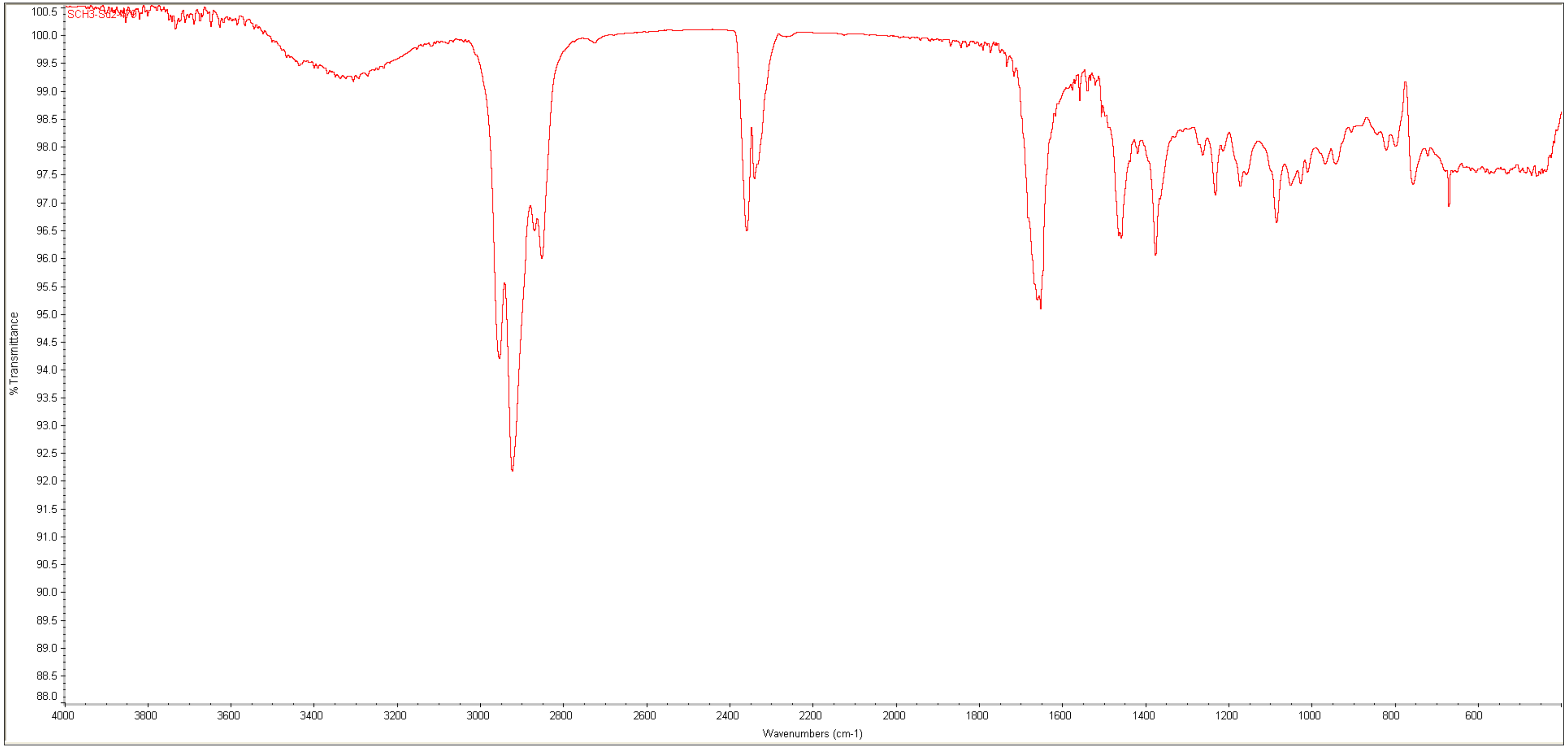

Figure S25. FT-IR spectrum of $CHCl_3$ background signal

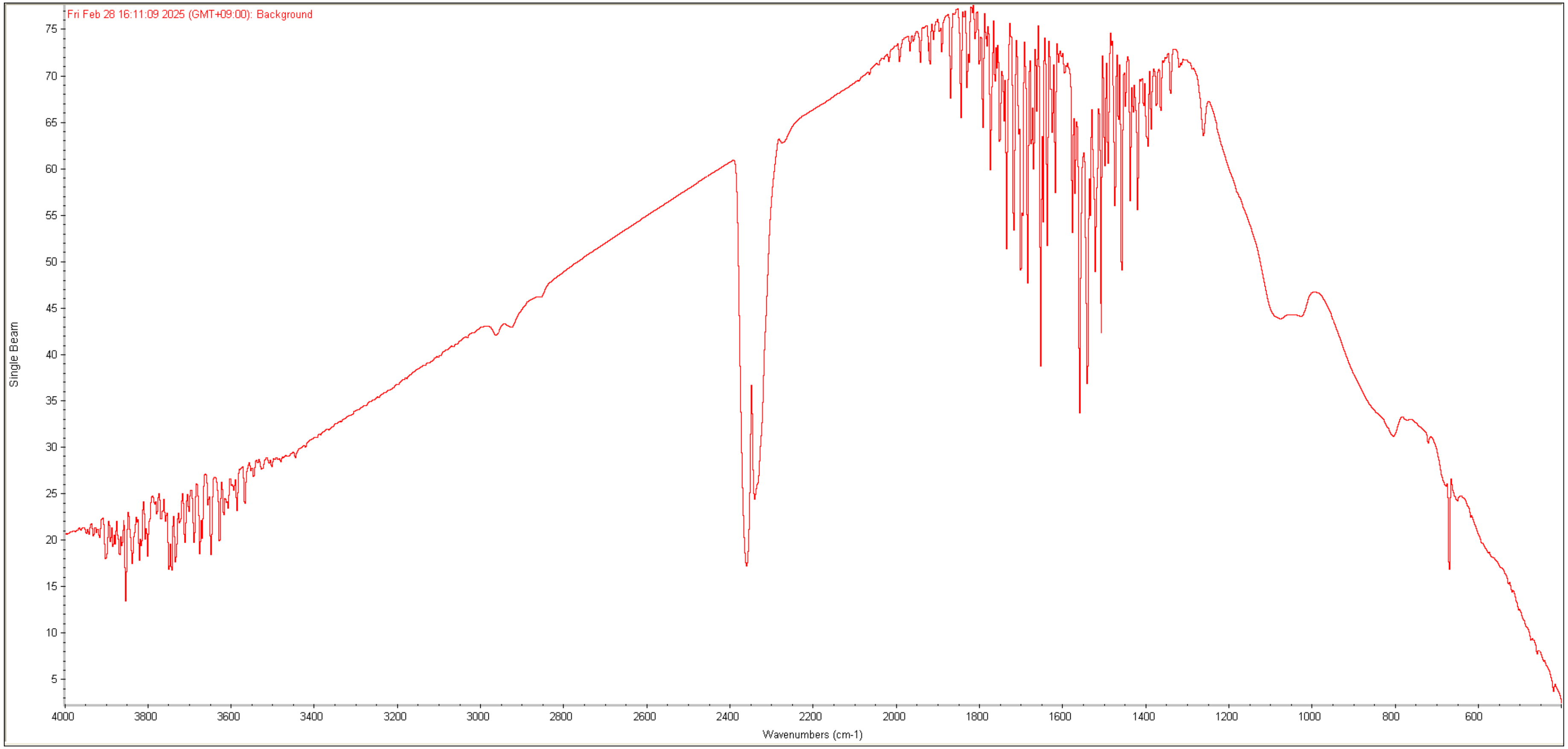

Table S1. ECD data of compounds **1–3**

Wavelength	Spirotryprostatin H (**1**)	Fumitremorgin O (**2**)	Fumitremorgin P (**3**)
500	0.0515194	-0.0331789	0.0276393
499	-0.0228753	-0.00669823	-0.0826267
498	-0.0383487	-0.0404587	-0.100553
497	-0.0475265	-0.0274842	-0.0840298
496	0.0035023	0.00529132	-0.0477981
495	0.0437235	0.039197	-0.0135075
494	0.0688012	0.0339694	-0.0390621
493	0.0702725	0.0205247	-0.0228784
492	0.0673654	0.0164148	-0.0114599
491	0.0473944	-0.00585372	-0.0172099
490	0.0394101	-0.00679431	-0.0261152
489	0.036808	-0.0156827	-0.0332629
488	0.0375848	-0.0254817	-0.0312583
487	0.0207606	-0.028261	-0.0356647
486	0.00953598	-0.0354326	-0.0474231
485	-0.00684709	-0.0378527	-0.0429636

484	-0.00677042	-0.0412663	-0.0446675
483	-0.0102591	-0.0380449	-0.0396753
482	-0.0080934	-0.0317416	-0.0306064
481	-0.00872823	-0.0257331	-0.027062
480	-0.0121482	-0.0158001	-0.0220208
479	-0.0098591	-0.0114992	-0.0259083
478	-0.0133786	-0.00807449	-0.0286673
477	-0.0181115	-0.010601	-0.0340594
476	-0.0148778	-0.0100548	-0.0328097
475	-0.0250293	-0.00929987	-0.0190684
474	-0.028841	-0.00144591	-0.015271
473	-0.0362512	-0.00630777	-0.0171472
472	-0.0451106	-0.0110253	-0.0249201
471	-0.0396323	-0.0158136	-0.0318961
470	-0.040684	-0.0195825	-0.0448973
469	-0.0436597	-0.0195019	-0.04718
468	-0.0408374	-0.0286266	-0.0570513
467	-0.0431713	-0.0249415	-0.0573201

466	-0.0356507	-0.0321144	-0.0616687
465	-0.0255492	-0.0345502	-0.0593561
464	-0.0260701	-0.03384	-0.060341
463	-0.0290147	-0.0255019	-0.0557039
462	-0.0337929	-0.0246365	-0.0526403
461	-0.041831	-0.0251405	-0.0592421
460	-0.0410352	-0.0304026	-0.0623096
459	-0.0455881	-0.0292332	-0.0531489
458	-0.0456564	-0.0281954	-0.0586276
457	-0.0553777	-0.0294342	-0.0522379
456	-0.0664223	-0.0384937	-0.0523734
455	-0.0579883	-0.0465441	-0.0587001
454	-0.0511257	-0.0370017	-0.051157
453	-0.0463544	-0.0305403	-0.049695
452	-0.0414334	-0.0285299	-0.0417269
451	-0.0355377	-0.031414	-0.0413642
450	-0.029992	-0.0266432	-0.0475252
449	-0.0286325	-0.0312239	-0.0528632

448	-0.0191223	-0.0285233	-0.0514594
447	-0.00701397	-0.0294671	-0.0516259
446	-0.00641438	-0.0308518	-0.0498405
445	-0.00919963	-0.0237142	-0.0444176
444	-0.0265472	-0.0299956	-0.041206
443	-0.0273797	-0.0289668	-0.0349623
442	-0.040973	-0.0237447	-0.0451611
441	-0.0446261	-0.0180437	-0.0377288
440	-0.0468032	-0.0255241	-0.0404583
439	-0.0577289	-0.0270196	-0.0441006
438	-0.0630462	-0.0221849	-0.0269244
437	-0.0648759	-0.0205753	-0.0161092
436	-0.0604756	-0.0251533	-0.0243458
435	-0.0557184	-0.0294809	-0.0246884
434	-0.0524331	-0.0289058	-0.0330335
433	-0.0510067	-0.016511	-0.0299508
432	-0.0380302	-0.0108045	-0.0288004
431	-0.0229898	-0.0158301	-0.0375445

430	-0.0194263	-0.0191079	-0.0387006
429	-0.021774	-0.0055527	-0.0364229
428	-0.0115493	-0.00131491	-0.0372394
427	-0.0038498	-0.00332151	-0.0345991
426	-0.00869888	0.00045858	-0.0273632
425	-0.014193	-0.0102988	-0.0257118
424	-0.0135917	-0.00262615	-0.026305
423	-0.0103583	-0.00145139	-0.0260953
422	-0.0232527	-0.00585332	-0.0250075
421	-0.0290581	-0.0160632	-0.0237523
420	-0.0375297	-0.0103183	-0.0216369
419	-0.0423848	-0.0059693	-0.0122379
418	-0.0375388	-0.00234581	-0.00629946
417	-0.0285217	-0.0053228	-0.0073047
416	-0.0329492	-0.000145343	-0.00867505
415	-0.0382381	-0.00104003	-0.0105333
414	-0.0339716	-0.00268762	-0.0218371
413	-0.0334166	0.00231456	-0.0225912

412	-0.0101939	0.00935921	-0.0244873
411	-0.00336303	0.0125183	-0.01845
410	0.00300153	0.00177577	-0.0277853
409	0.0142453	0.00316609	-0.0361253
408	0.0334072	0.0171531	-0.0324479
407	0.049534	0.0261186	-0.0173122
406	0.0534935	0.0242098	-0.020343
405	0.0618469	0.0135906	-0.0151855
404	0.0690276	0.00448782	-0.0134519
403	0.0580402	-0.00580359	-0.0199842
402	0.0471354	-0.0115071	-0.0224012
401	0.0511609	-0.0192758	-0.0262431
400	0.0485419	-0.0293851	-0.020407
399	0.0550164	-0.0323072	-0.023682
398	0.0517665	-0.0223334	-0.0151823
397	0.0574565	-0.0246011	-0.0228098
396	0.0398408	-0.023377	-0.0246008
395	0.0302554	-0.0330149	-0.0451489

394	0.0169613	-0.0255086	-0.0443173
393	0.00742372	-0.0361548	-0.0463616
392	0.00397852	-0.0361238	-0.0523486
391	-0.00306967	-0.0346361	-0.0592093
390	-0.000485218	-0.0312004	-0.0589343
389	-0.00205512	-0.0319943	-0.0575824
388	0.00194959	-0.0222439	-0.0581362
387	0.00755806	-0.0140499	-0.057594
386	0.00628684	-0.0207633	-0.0490673
385	0.00502526	-0.0264075	-0.0517854
384	0.00204733	-0.0279399	-0.0547726
383	0.00108923	-0.0311839	-0.0474353
382	0.0127913	-0.0331505	-0.0477613
381	0.0273131	-0.0358744	-0.0454293
380	0.0331699	-0.0441644	-0.0396569
379	0.0412188	-0.0415843	-0.0458984
378	0.043146	-0.0378101	-0.0335027
377	0.0594357	-0.0391036	-0.0433534

376	0.0477117	-0.0333044	-0.0313482
375	0.0490746	-0.0383053	-0.0284701
374	0.0497037	-0.036062	-0.0281993
373	0.0559073	-0.0502825	-0.0412078
372	0.050952	-0.062096	-0.0452211
371	0.050475	-0.0741118	-0.0480508
370	0.0541634	-0.0755475	-0.0493385
369	0.0566302	-0.0816201	-0.0517747
368	0.0609152	-0.0778705	-0.0386854
367	0.0657312	-0.0779924	-0.0368606
366	0.0609025	-0.0806973	-0.0376432
365	0.0616373	-0.102513	-0.0537356
364	0.0552947	-0.116381	-0.0597752
363	0.0566621	-0.112173	-0.0674501
362	0.0540507	-0.124781	-0.0705393
361	0.0455068	-0.132243	-0.0706872
360	0.0380273	-0.148385	-0.0737015
359	0.0294999	-0.160834	-0.0837543

358	0.0166988	-0.168639	-0.0896742
357	0.0068054	-0.165193	-0.0956853
356	-0.00337008	-0.166115	-0.0883713
355	0.00535806	-0.170809	-0.0922913
354	0.00927503	-0.170605	-0.0782935
353	0.00931948	-0.185556	-0.083864
352	-0.00414098	-0.19097	-0.0983683
351	-0.00919751	-0.208945	-0.104904
350	-0.00421225	-0.213317	-0.104909
349	0.00743656	-0.214798	-0.0968368
348	0.00993325	-0.209456	-0.0949125
347	0.0192726	-0.208266	-0.0967375
346	0.0128953	-0.203666	-0.0867518
345	0.0161536	-0.207444	-0.0813293
344	0.0188227	-0.219519	-0.0843803
343	0.022152	-0.230192	-0.0919084
342	0.0127612	-0.234786	-0.0892666
341	0.00688804	-0.242663	-0.0765658

340	0.00993813	-0.259266	-0.0827009
339	-0.000729893	-0.258101	-0.0817134
338	0.000239995	-0.26694	-0.0885374
337	0.00239443	-0.272691	-0.0891418
336	0.0166725	-0.278874	-0.0868258
335	0.0240765	-0.285736	-0.0829705
334	0.0258903	-0.297488	-0.0856579
333	0.0190878	-0.309115	-0.0833565
332	0.0126888	-0.323901	-0.0857507
331	0.0112869	-0.33083	-0.0890348
330	0.0171066	-0.337379	-0.0888915
329	0.017196	-0.343805	-0.0849124
328	0.00014633	-0.339457	-0.0855929
327	-0.00678909	-0.328528	-0.0895852
326	-0.0272407	-0.289916	-0.0909509
325	-0.037436	-0.235247	-0.090916
324	-0.0589521	-0.119506	-0.0760083
323	-0.0771649	0.0390334	-0.0715881

322	-0.0959716	0.261891	-0.0798299
321	-0.110822	0.568988	-0.0805289
320	-0.124209	0.959625	-0.0827547
319	-0.154065	1.44233	-0.0645291
318	-0.166404	2.01655	-0.0641583
317	-0.191448	2.67224	-0.0399444
316	-0.213895	3.38803	-0.0077766
315	-0.245767	4.1538	0.0372444
314	-0.276569	4.95561	0.0833505
313	-0.325731	5.7717	0.148885
312	-0.369065	6.60592	0.226918
311	-0.425846	7.41629	0.317569
310	-0.49332	8.18171	0.406375
309	-0.595606	8.89569	0.511136
308	-0.725288	9.52595	0.614727
307	-0.888435	10.0603	0.71769
306	-1.12113	10.5049	0.822982
305	-1.41483	10.8276	0.927383

304	-1.79238	11.0452	1.02903
303	-2.23775	11.1409	1.1133
302	-2.7518	11.1607	1.19922
301	-3.28598	11.0662	1.26767
300	-3.82102	10.9015	1.31809
299	-4.365	10.6789	1.35956
298	-4.89508	10.4002	1.38822
297	-5.41942	10.0892	1.4051
296	-5.89368	9.75251	1.40117
295	-6.33726	9.40796	1.39169
294	-6.70564	9.08182	1.38388
293	-7.00644	8.79545	1.3592
292	-7.23796	8.58802	1.32925
291	-7.38643	8.49522	1.29717
290	-7.48279	8.55033	1.27541
289	-7.50685	8.75255	1.24234
288	-7.48682	9.13625	1.22146
287	-7.4536	9.67592	1.22529

286	-7.41173	10.3419	1.24226
285	-7.37279	11.101	1.2806
284	-7.34872	11.9166	1.34965
283	-7.31552	12.7183	1.42936
282	-7.28061	13.4997	1.51979
281	-7.21806	14.2252	1.62646
280	-7.16218	14.8817	1.74025
279	-7.13398	15.3976	1.84815
278	-7.12075	15.8002	1.94771
277	-7.1256	16.0777	2.04263
276	-7.1376	16.2046	2.12606
275	-7.16381	16.1676	2.17624
274	-7.19165	15.9916	2.2203
273	-7.22186	15.6678	2.23753
272	-7.27189	15.2312	2.24446
271	-7.3097	14.6942	2.21587
270	-7.3556	14.1087	2.16228
269	-7.36685	13.4864	2.09421

268	-7.38522	12.8463	2.00738
267	-7.39713	12.1978	1.89751
266	-7.38139	11.5548	1.7873
265	-7.36315	10.8794	1.67345
264	-7.31555	10.1667	1.55389
263	-7.25252	9.40849	1.4297
262	-7.18711	8.59105	1.29891
261	-7.075	7.71839	1.15823
260	-6.9417	6.79897	1.01629
259	-6.77839	5.84452	0.859791
258	-6.57647	4.86141	0.694532
257	-6.32631	3.86331	0.537808
256	-6.00581	2.90197	0.376852
255	-5.5821	1.96683	0.214497
254	-5.01869	1.10709	0.039015
253	-4.27735	0.326828	-0.132302
252	-3.31643	-0.30683	-0.289564
251	-2.0993	-0.85617	-0.442505

250	-0.592244	-1.29906	-0.594302
249	1.19047	-1.66186	-0.745699
248	3.21937	-1.99935	-0.897089
247	5.45019	-2.32376	-1.04379
246	7.84306	-2.63582	-1.19442
245	10.2767	-2.9882	-1.3493
244	12.6846	-3.34753	-1.51174
243	14.9245	-3.77898	-1.68068
242	16.9347	-4.32906	-1.86523
241	18.5968	-4.97724	-2.06769
240	19.7641	-5.67607	-2.28099
239	20.3604	-6.44049	-2.49216
238	20.3444	-7.22055	-2.71359
237	19.6918	-8.06828	-2.94393
236	18.3773	-8.91918	-3.17848
235	16.5474	-9.67318	-3.43042
234	14.3579	-10.4058	-3.69884
233	11.8001	-10.9786	-3.91909

232	9.08874	-11.3259	-4.12304
231	6.31933	-11.5144	-4.24766
230	3.66123	-11.449	-4.25804
229	1.1667	-11.1974	-4.27833
228	-1.07479	-10.7354	-4.16637
227	-2.9022	-10.1535	-3.97005
226	-4.28901	-9.54769	-3.73293
225	-5.12723	-8.85966	-3.36975
224	-5.46375	-8.16149	-3.01321
223	-5.30439	-7.47817	-2.62136
222	-4.61824	-6.77966	-2.17468
221	-3.50318	-6.24252	-1.72067
220	-2.032	-5.79314	-1.32395
219	-0.327158	-5.44574	-0.932821
218	1.60389	-5.14068	-0.6459
217	3.54797	-4.87285	-0.38293
216	5.44396	-4.65946	-0.141435
215	7.30192	-4.43745	-0.00862056

214	8.89238	-4.15876	0.0706584
213	10.073	-3.80082	0.118058
212	10.8044	-3.39904	0.131718
211	11.1049	-2.87601	0.140197
210	10.8894	-2.24063	0.173447
209	10.1894	-1.51334	0.182406
208	9.15644	-0.549291	0.16509
207	8.00456	0.357345	0.0488464
206	6.71796	1.26533	0.0931735
205	5.34771	2.07938	0.0831468
204	4.00919	2.68765	0.0920887
203	2.74837	3.20071	0.10331
202	1.56606	3.4937	0.00258637
201	0.589942	3.66736	0.025649
200	-0.144118	3.85504	0.0668802
199	-0.714576	3.68009	0.00561962
198	-0.8424	3.23855	-0.0879994
197	-0.643476	2.60158	-0.156912

196	-0.556906	1.7945	-0.111098
195	-0.758569	0.892404	-0.00431428
194	-1.19008	0.308377	0.176074
193	-1.21895	0.186211	0.164506
192	-0.927674	0.617164	0.673256
191	-0.612559	1.26009	1.70918
190	-0.857733	1.07478	1.02934

Table S2. NMR correlation data for compounds **1–3** in DMSO-d_6

Pos.	1				2				3			
	δ_C[a], mult.	δ_H[b] (*J* in Hz)	COSY	HMBC	δ_C[a], mult.	δ_H[b] (*J* in Hz)	COSY	HMBC	δ_C[a], mult.	δ_H[b] (*J* in Hz)	COSY	HMBC
1-NH										10.44, s		
2	182.3, qC				132.3, qC				131.1, qC			
3	55.9, qC				103.3, qC				106.5, qC			
3a	119.8, qC				121.1, qC				121.5, qC			
4	126.8, CH	6.99, d (8.3)	5	3, 6, 7a	118.4, CH	7.42, d (8.3)	5	3, 6, 7a	117.7, CH	7.56, d (8.7)	5	3, 7a
5	106.7, CH	6.50, dd (8.3, 2.4)	4, 7	3a, 6, 7	108.5, CH	6.69, dd (8.3, 2.2)	4, 7	4, 7	105.6, CH	6.76, d (8.7)	4	3a, 7
6	159.8, qC				155.3, qC				152.2, qC			
7	96.5, CH	6.40, d (2.4)		3a, 6, 7a	93.4, CH	6.80, d (2.2)		4, 5, 6	111.9, qC			
7a	142.8, qC				137.0, qC				136.5, qC			
8	41.2, CH_2	α 2.35, d (15.0) ; β 2.89, d (15.0)		2, 3, 3a, 9, 18	29.4, CH_2	α 3.08, d (16.0) ; β 3.40, d (16.0)		2, 3, 3a, 9	67.8, CH	5.51, s		2, 3
9	89.4, qC				82.9, qC				83.3, qC			
11	168.2, qC				164.9, qC				166.2, qC			
12	60.1, CH	4.44, m	13	11, 13	57.9, CH	4.37, m	13	13[c]	58.2, CH	4.41, m	13	14, 17
13	27.8, CH_2	α 1.89, m ; β 2.22, m	12, 14		28.5, CH_2	α 1.85, m ; β 2.27, m	12, 14		28.5, CH_2	α 1.87, m ; β 2.29, m	12, 14	

14	22.7, CH_2	1.90, m	13, 15		22.1, CH_2	1.86, m	13, 15		22.0, CH_2	1.89, m	13, 15	
15	44.8, CH_2	3.43, m	14		44.7, CH_2	3.45, m	14		44.9, CH_2	3.45, m	14	
17	164.3, qC				169.9, qC				170.2, qC			
18	60.9, CH	4.79, d (9.0)	19	2, 3, 8, 19, 20	40.7, CH	5.93, d (9.8)	19	19, 20	48.7, CH	5.83, d (9.5)	19	2, 3, 9,19, 20
19	121.8, CH	4.92, d (9.0)	18	21, 22	123.1, CH	4.82, d (9.8)	18	21[c]	124.0, CH	4.78, d (9.5)	18	21, 22
20	135.2, qC				132.9, qC				133.0, qC			
21	17.7, CH_3	1.16, s		19, 20, 22	17.7, CH_3	1.91, s		19, 20, 22	18.4, CH_3	1.92, s		19, 20, 22
22	25.1, CH_3	1.49, s		19, 20, 21	25.1, CH_3	1.59, s		19, 20, 21	25.4, CH_3	1.57, s		19, 20, 21
23					41.0, CH_2	4.54, m; β 4.64, m	24	25[c]	23.4, CH_2	3.54, m / 3.41, m	24	6, 7, 7a, 24, 25
24					120.4, CH	4.98, m	23	26[c], 27[c]	123.1, CH	5.20, m	23	26, 27
25					133.7, qC				130.2, qC			
26					18.0, CH_3	1.85, s		24, 25, 27	17.9, CH_3	1.76, s		24, 25, 27
27					25.2, CH_3	1.66, s		24, 25, 26	25.2, CH_3	1.62, s		24, 25, 26
6-OMe	55.2, CH_3	3.72, s		6	55.0, CH_3	3.76, s		6	56.4, CH_3	3.76, s		6
8-OH										4.76, s		2, 3, 3a, 7a
9-OH		7.42, s		9, 17		6.53, br s				4.09, s		

[a] 400 MHz for ^1H NMR. [b] 100 MHz for ^{13}C NMR. [c] Weak HMBC signals.

Figure S26. LC chromatograms of L and D-FDLA derivatives of proline from spirotryprostatin H (**1**)

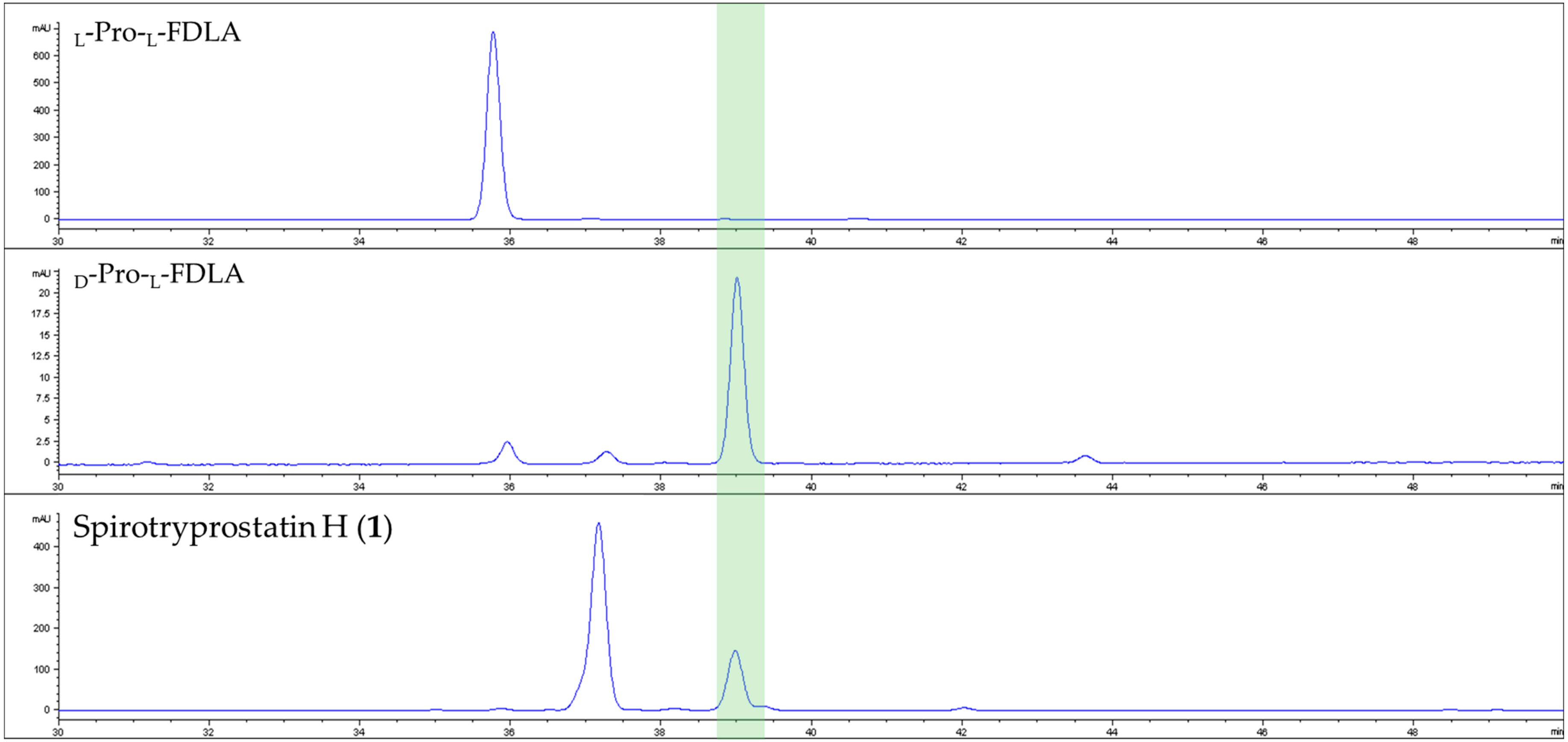

Figure S27. LC chromatograms of L and D-FDLA derivatives of proline from fumitremorgins O (**2**) and P (**3**)

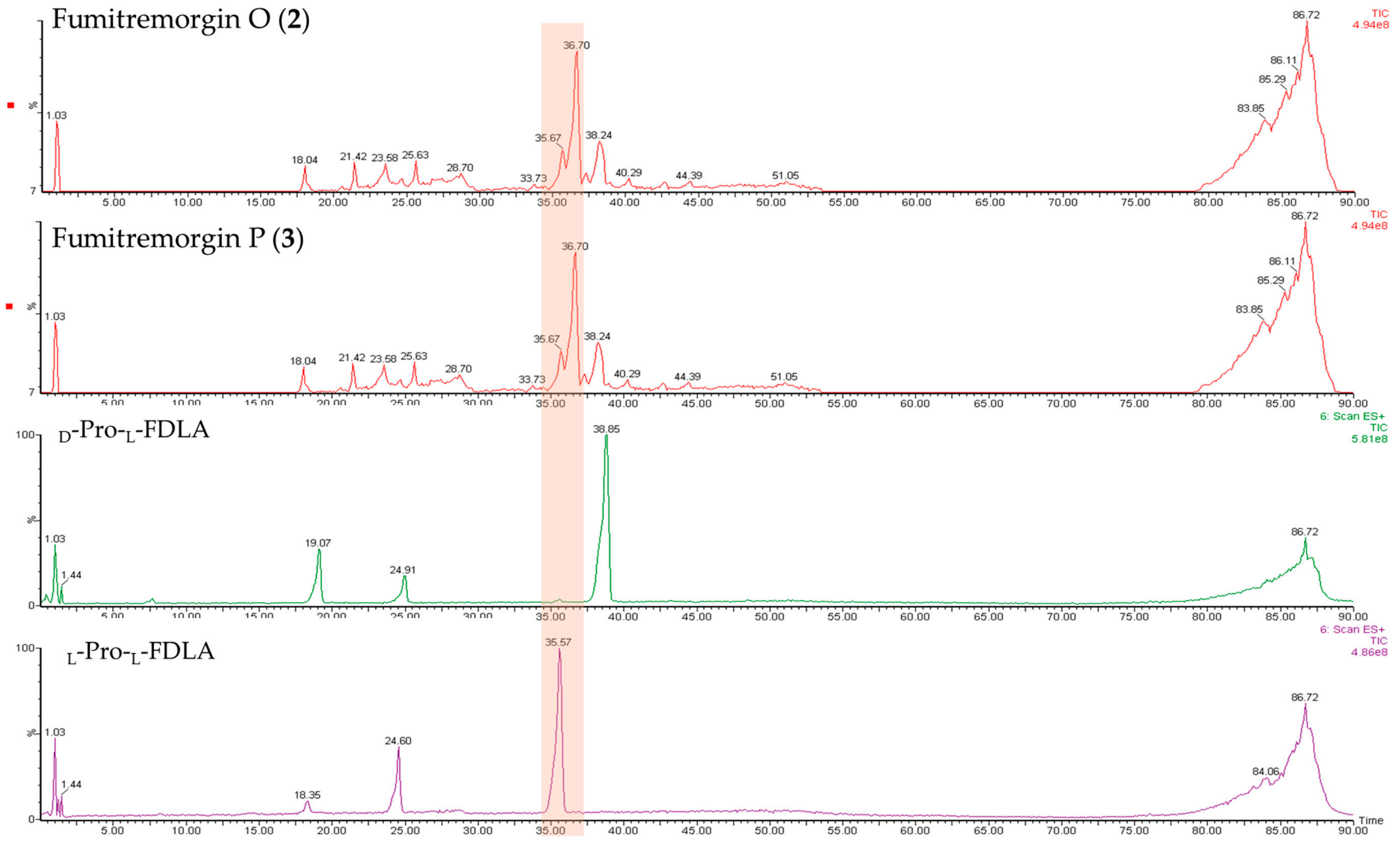

Figure S28. β-tubulin gene sequence of strain SCH3-Sd2

TCTGGTACGTGCGGCGTCCGGACGAAACTTTTCGCGTCGGGTATCAATTGACAACAAATTGCTAACTGGGAGATAGGCAA

ACCATCTCTGGCGAGCACGGTCTCGATGGTGATGGACAGTAAGTTTTATGTGAAGGATTCTATCATGGTGGATTGGCTGTC

TGACATGTTTCTAGGTACAATGGTACCTCCGACCTCCAGCTCGAGCGTATGAACGTCTACTTCAACCATGTGAGTACAATG

GCTTTGCAACCTGTTTCTCGTACATCTTCTAATCAAATCTTTTTCCTTGTCAATCTAGGCCAGCGGTGATAAGTACGTTCCC

CGTGCCGTTCTCGTCGATTTGGAGCCTGGTACCATGGACGCCGTCCGCTCCGGTCCTTTCGGCAAGCTCTTCCGCCCCGAT

AACTTCGTCT
